# Livelihood and Dietary Transitions in Subsistence Populations of Northern Laos: Insights From Carbon and Nitrogen Stable Isotope Analysis

**DOI:** 10.1002/ajpa.70326

**Published:** 2026-07-31

**Authors:** Ziyang Li, Mihoko Kibe, Yuki Mizuno, Masafumi Saitoh, Kohei Yamazaki, Hiroaki Masuoka, Satoko Kosaka, Kazumi Natsuhara, Kazuhiro Hirayama, Nouhak Inthavong, Sengchanh Kounnavong, Minoru Yoneda, Shinsuke Tomita, Masahiro Umezaki

**Affiliations:** ^1^ Department of Human Ecology, School of International Health, Graduate School of Medicine The University of Tokyo Tokyo Japan; ^2^ The University Museum The University of Tokyo Tokyo Japan; ^3^ Laboratory for Symbiotic Microbiome Sciences RIKEN Center for Integrative Medical Sciences Yokohama Kanagawa Japan; ^4^ Faculty of Nursing Toho University Tokyo Japan; ^5^ Laboratory of Veterinary Public Health, Graduate School of Agricultural and Life Sciences The University of Tokyo Tokyo Japan; ^6^ Lao Tropical and Public Health Institute, Ministry of Health Vientiane Capital Lao PDR; ^7^ Graduate School of Environmental Studies Nagoya University Nagoya Japan

**Keywords:** Laos, market integration, Southeast Asia, stable isotopes, subsistence transition

## Abstract

**Objectives:**

Rural populations in northern Laos have experienced dramatic socioeconomic transformation driven by national policies and regional economic integration, substantially influencing their self‐sufficient livelihoods. To investigate how these changes have manifested in local subsistence contexts, we compared hair carbon and nitrogen isotope values between two populations representing swidden‐ and paddy‐farming systems, and assessed how isotopic variations reflect the differences in subsistence practices and degrees of livelihood transformation. By analyzing commonly consumed foodstuffs, we provide the first isotopic dataset of foods from northern Laos and baselines for interpreting human tissue isotope values.

**Methods:**

Scalp hair and food samples and sociodemographic data were collected from the study villages. In total, 175 hair and 69 food samples were analyzed. Multiple linear regression analyses were used to examine associations with subsistence/economic and dietary variables. Rice–hair isotopic offsets were calculated to qualitatively assess dietary patterns.

**Results:**

Hair *δ* ^15^N was higher in the paddy‐farming village than in the swidden‐farming village. In the swidden‐farming village, individuals who shifted to paddy cultivation showed higher *δ* ^15^N and lower *δ* ^13^C values than those who primarily practiced swidden cultivation. In the paddy‐farming village, individuals with greater market involvement exhibited higher hair *δ* ^13^C values. The rice–hair offsets suggested elevated market food consumption among paddy field cultivators.

**Discussion:**

The differences in hair isotope values between (sub)populations can be attributed to isotopic variations in rice, the staple food, and to the differences in market participation. Market integration can lead to an increase in hair *δ* ^13^C, but the overall isotopic outcome is highly context dependent.

## Research Highlights

- An isotopic snapshot of two rural populations in northern Laos undergoing a transition from subsistence to market economies.
- Isotopic variation mirrors socioeconomic differences in livelihood strategies and market engagement.
- Dietary isotope signals in subsistence populations may converge toward those of urban populations as globalization progresses.

## Graphical abstract

Conceptual Framework: Livelihood, diet, and hair isotope values.

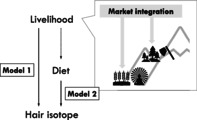

## 1 Introduction

Laos is among the last regions on the planet to witness the degradation of foraging, which was once the most common and long‐lasting subsistence strategy in human history (cf. Stephens et al. 2019). Prior to the economic reforms of the Lao People's Democratic Republic (Lao PDR) in the late twentieth century, subsistence was primarily characterized by a complex mix of foraging (including hunting, gathering, and fishing) and multiple farming systems (Rigg 2005/2012b; Savada 1995). However, over the past two decades, the rapid expansion of market‐oriented economic activities has increasingly taken over the space once held by traditional self‐sufficient livelihoods (Rigg 2005/2012a; Thongmanivong and Fujita 2006).

This transition in Laos is part of a global trend of market integration driving the restructuring of people's livelihood strategies, defined as a dynamic ensemble of subsistence and economic activities. As these practices directly shape opportunities for food acquisition, the changes can be traced through stable isotopic compositions of diets or human tissues (e.g., hair and nail keratin and bone collagen), which can be used as proxies for diet (DeNiro and Epstein 1978, 1981; O'Brien 2015; Reitsema 2015). Stable isotopes thus provide an “integrated signal of human utilization and manipulation of local food webs” (Bird et al. 2021, 4), enabling the reconstruction of dietary and ecological shifts associated with socioeconomic transformations. For example, studies using carbon and nitrogen isotope values have shown that dietary shifts driven by market integration are often uneven and socially differentiated. Nardoto et al. (2020) found that the nutrition transition had not reached all remote populations in Brazil, while Nash et al. (2012) observed variation in traditional and market food intake across sociodemographically differentiated groups in indigenous communities of Southwest Alaska. In comparing several populations from Brazil and the United States, Nardoto et al. (2006) concluded that carbon and nitrogen isotope values continued to reflect regional dietary practices despite increasing dietary homogenization under transnational food networks. At a global scale, Bird et al. (2021) demonstrated a substantial reduction in dietary isotopic niche breadth in industrialized societies compared with ancient and modern subsistence populations, underscoring how stable isotopes can illuminate dietary shifts associated with livelihood transitions.

**FIGURE 1:**
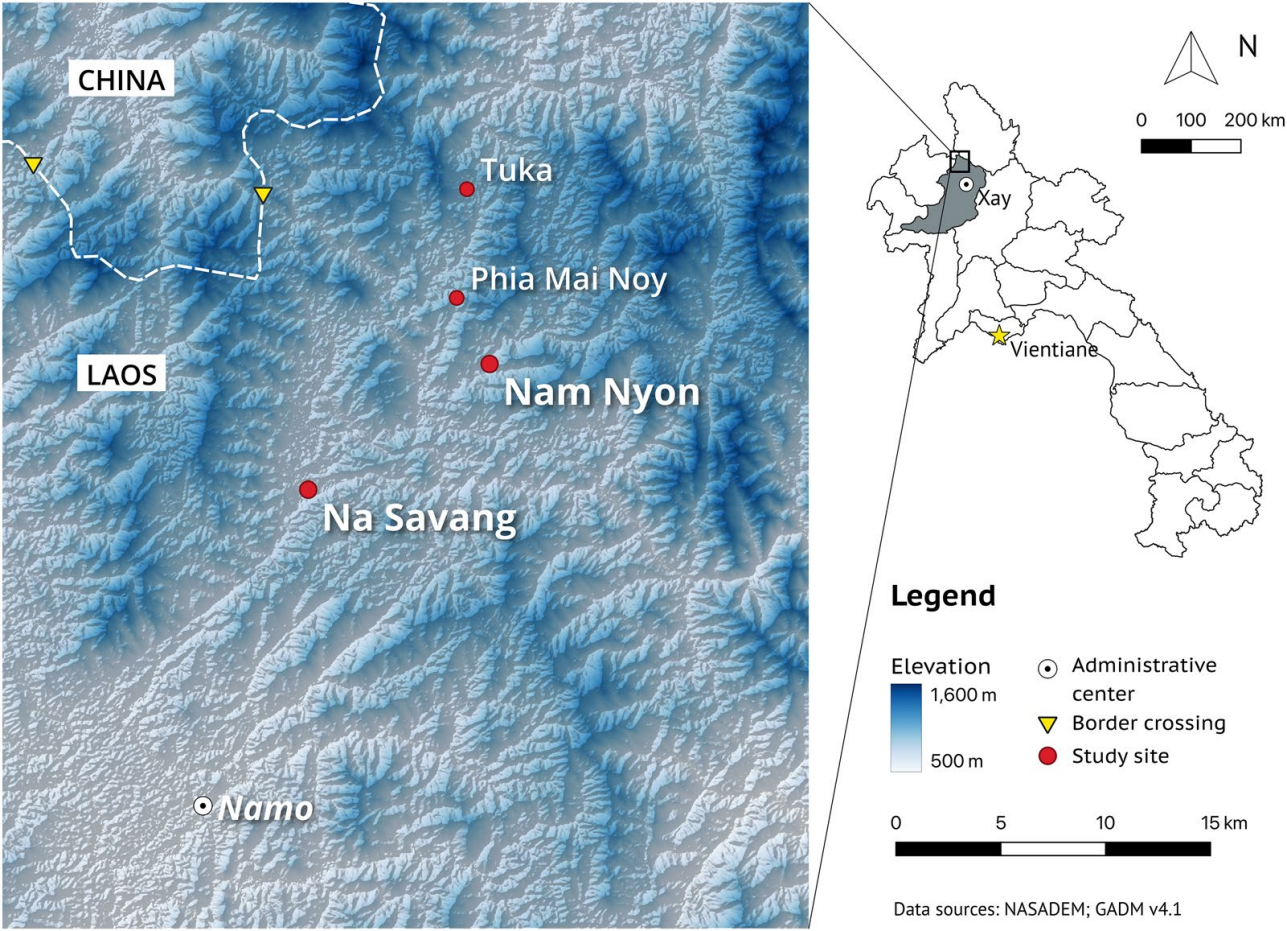
Location of the study area in Oudomxay Province, northern Laos. The inset map shows the location of Oudomxay Province (blue‐gray), as well as Muang Xay, the provincial capital, and Vientiane, the national capital of Lao People's Democratic Republic. The main map on the left shows the location of Namo, the district center, and the study villages. Na Savang and Nam Nyon (bold) are the main study villages where hair and food samples were collected. Tuka and Phia Mai Noy are upland villages where additional food samples were collected. The main map is based on NASADEM elevation data (NASA JPL 2021). Administrative boundaries in the main and inset maps are from GADM v4.1 (GADM 2022).

This study focused on rural populations in Oudomxay Province, Lao PDR (Figure 1), which borders China's Yunnan Province to the north and forms part of the Southeast Asian Massif (Michaud 1997; Michaud et al. 2016). Also known as Zomia (Scott 2009; van Schendel 2002), this region is characterized by significant ethnolinguistic heterogeneity and a long‐standing lowland–upland dichotomy in subsistence regimes that is closely tied to ethnolinguistic divisions (Chazée 1999; McKinnon and Michaud 2000). Lowland subsistence is based on the intensive cultivation of wet rice (usually the glutinous variety, *Oryza sativa* var. *glutinosa*) in plains and valleys, whereas upland economies are typified by swidden‐based dry rice cultivation on slopes and the extensive use of non‐timber forest products (NTFPs). Despite these differences, in rural Laos, both patterns rely on rice farming and the use of wild resources as vital components of their traditional livelihoods (Food and Agriculture Organization of the United Nations 2013; Rigg 2006, 2005/2012b).

The study area has undergone profound socioeconomic transformation since the early 2000s—a process driven by national policies and regional economic integration, particularly the continued expansion of the Chinese economy (Manorom et al. 2011; Onphanhdala 2022; Rigg 2005/2012a; Yokoyama and Ochiai 2008). To examine how these changes have manifested in different subsistence contexts, we conducted a stable isotope analysis of human hair and local food samples from two rural communities in Namo District, Oudomxay Province, representing lowland and upland settings. The objectives of this study were (i) to compare the hair carbon and nitrogen stable isotopic compositions between lowland and upland populations and (ii) to explore the extent to which intrapopulation variation reflects the subsistence transition, mediated by shifts in dietary practices. We also analyzed food samples from the study area to characterize the isotopic composition of locally consumed resources, including wild edible plants, cultivated crops, animal‐derived foods, and packaged foods purchased from markets. In doing so, this study furnishes the first isotopic dataset of foods from northern Laos and provides essential baselines for interpreting human tissue isotope values.

## 2 Materials and Methods

### 2.1 Study Populations

#### 2.1.1 Nam Nyon: Upland Livelihoods Transitioning From Shifting to Permanent Farming

Nam Nyon is a small and relatively isolated village situated in a narrow tributary valley. It is about 2.5 h by motorbike from the nearest market in Namo and is not accessible by road during the rainy season. Local livelihoods have traditionally depended on swidden‐based shifting cultivation and foraging. Daily diet is dominated by rice, which provides approximately 87% of the total energy and 57%–59% of protein intake (Kibe et al. 2022), supplemented with a wide variety of wild and cultivated plants. Animal protein is only consumed occasionally, mainly from small aquatic animals and wild game obtained through fishing and hunting (Kibe et al. 2022).

The village had a population of 252 residents as of 2019 (Kibe et al. 2022). Most residents are Kongsat and Phunyot, two Sino‐Tibetan‐speaking ethnic groups. The Sino‐Tibetan language family remains dominant among the upland peoples. However, today, both the Kongsat and Phunyot are extremely sparse in number. Indeed, Nam Nyon is the only settlement where they constitute the majority of the population. According to locals, the Kongsat founded the present village in 1981. The Phunyot, who used to settle deeper in the forests, moved to Nam Nyon around 2008, due to national programs promoting the resettlement of upland villages to lowland areas to facilitate the switch from shifting cultivation to permanent farming, improve access to markets and government services, and accelerate social and cultural integration (Baird and Shoemaker 2007; Evrard and Goudineau 2004).

In 2004, 15 of the 17 Kongsat households in the village practiced swidden farming, and only 2 had paddy fields. However, by 2018, 57% of households owned paddy fields (Kibe et al. 2022). Increased paddy field ownership was paralleled by a surge in cash cropping. Tobacco and sugarcane are the dominant commercial crops and are cultivated in permanent paddies and swiddens, respectively. As such, the village is transitioning from shifting to permanent farming. This transition in subsistence practices reflects growing market involvement and declining mobility associated with the incorporation of upland communities into market and state structures. This process is a marked departure from the mobility and relative autonomy historically attributed to upland Zomia societies (Scott 2009).

#### 2.1.2 Na Savang: Lowland Paddy Farming Under Market Integration

Na Savang lies in an intermontane basin about 800 m above sea level. The village is a two‐to‐three‐hour drive from the provincial capital and one hour from the Laos–China border. The village had a population of 799 as of 2019 (Tomita 2020). Most residents are ethnic Yang, who are speakers of a Tai‐Kradai language, the dominant lowland ethnolinguistic family.

The village was once the political and economic center of a small chiefdom (*muang*) under the Kingdom of Luang Prabang (1707–1893). Local livelihood predominantly relies on wet glutinous rice, which accounts for approximately 85% of residents' caloric intake (Tomita et al. 2015), supplemented with self‐grown vegetables and various aquatic and forest resources. Villagers raise livestock primarily for trade and ritual purposes, rather than for daily consumption. Prior to the 2010s, cash income from economic activities was mainly used to cover occasional expenses, such as weddings or medical care (Tomita 2020).

According to field interviews and observations by one of the authors (S.T.), who has conducted longitudinal fieldwork in the village for over two decades, these patterns have shifted in recent years. While paddy farming and rice self‐sufficiency are sustained, the importance of cash crops—mostly for the Chinese market—has gradually increased. The village was electrified in 2010, enabling the use of mobile phones. Cash crop cultivation has become commonplace, while the time allocated to foraging activities has declined. Household ownership of motorcycles and cars has also increased, and a small market selling fresh meat and eggs, packaged foods, and daily necessities has been established nearby. Some villagers have started small‐scale commerce besides farming. Village life is rapidly being permeated by the market economy in a changing socioeconomic environment where currency is becoming increasingly important.

### 2.2 Data and Sample Collection

Sociodemographic and anthropometric data and scalp hair samples were collected from Na Savang in August 2018 (the rainy season) and from both Na Savang and Nam Nyon in March 2019 (the dry season). A total of 294 individuals aged ≥ 18 years from the two villages participated in the surveys, including 133 from Na Savang who took part in both survey rounds (total data and sample collections: *n* = 427). Participants were recruited with the assistance of village leaders and through loudspeaker announcements to cover as broad a segment of the village population as possible (Kibe et al. 2024; Mizuno et al. 2023; Sekiya et al. 2024). All participants provided written informed consent before data and sample collection.

The participants were interviewed using a questionnaire covering their gender, ethnicity, birth date (or year), economic activities (i.e., crop production, trades, and other income‐generating activities), household possessions (i.e., landholding [ha], roofing material, and ownership of durable goods), and consumption frequency (i.e., days of intake in the past week) of 28 food items selected a priori based on field experience in the study area to represent common local foods or food categories. Age was calculated by rounding the difference between the survey date and birth date (or year). Occupation was classified based on reported economic activities. Specifically, participants were categorized as “farming only” if they engaged only in crop production, “non‐farming only” if they engaged only in non‐crop economic activities, and “both” if they engaged in both. For rice self‐sufficiency, participants who produced rice without purchase or sale were classified as “self‐sufficient,” whereas those who produced and sold rice were classified as “surplus” (Kibe et al. 2024).

Hair samples were collected from all participants during the 2018 and 2019 surveys by cutting a lock of 20–30 strands of hair with stainless steel scissors as close to the scalp as possible. Hair strands were adhered to the root end with masking tape and stored in plastic pockets until the analysis. All participants provided hair samples, but only those from male subjects (*n* = 175, including 102 samples from 51 individuals sampled in both seasons) were analyzed to reduce variation unrelated to the livelihood transition of interest. Female body *δ* ^15^N can be influenced by metabolic changes during pregnancy and lactation (Fuller et al. 2004, 2005), as well as by culturally prescribed food taboos during the prenatal and postpartum periods. In northern Laos, such taboos are common among women and may involve avoidance of particular meats, fruits, and vegetables, with practices varying by ethnic group and locality and typically lasting from about one to several months, and sometimes years (Holmes et al. 2007; Smith et al. 2022). By contrast, comparable restrictions applying to men appear to be less frequently reported. Our field observations and interviews likewise indicated the presence of food taboos affecting pregnant and/or postpartum women in both study villages. For instance, pregnant women in Na Savang were reportedly expected to avoid the meat of albino buffalo, while postpartum women in Nam Nyon avoided any kinds of meat for several months after childbirth. Outside these life stages, however, sex differences in routine dietary patterns within households were limited (Kibe et al. 2022).

Food samples were collected from four villages in Namo District in 2023, namely, Na Savang, Nam Nyon, Tuka, and Phia Mai Noy (Figure 1). Tuka and Phia Mai Noy are upland villages inhabited by the Akha ethnic group and were included to enhance the diversity and representativeness of the food isotopic baseline. The samples comprised commonly consumed plant foods (e.g., rice and wild edible plants), animal foods (e.g., chicken and fish), and packaged convenience foods available in local stores or markets (e.g., instant noodles, beverages, and snacks), corresponding to 18 of the 28 items and/or categories included in the dietary questionnaire.^1^ Details of the samples analyzed are provided in Table S1. The samples were dried immediately after collection and stored and transported under frozen conditions to the University of Tokyo, where they were kept at −20°C until analysis.

### 2.3 Stable Isotope Analysis

#### 2.3.1 Scalp Hair Samples

To investigate dietary seasonality, a single 1‐cm scalp‐proximal segment was cut from each hair sample, representing about 1 month of dietary intake prior to sampling (Cooper 2015; Society of Hair Testing 2004). The cleaning of the samples followed a slightly modified protocol based on previously published methods (O'Connell et al. 2001; O'Connell and Hedges 1999). The samples were rinsed ultrasonically in Milli‐Q water for 10 min, followed by a 30‐min ultrasonic treatment with methanol–chloroform (2:1, v/v) to remove lipids, shampoos, and other external contaminants. Residual solvents were eliminated by two 5‐min ultrasonic rinses in Milli‐Q water. The cleaned samples were dried at 60°C, and 0.4 ± 0.05 mg of each sample were weighed into tin capsules using a microbalance.

Carbon and nitrogen isotope ratios of the hair samples were determined using elemental analyzer/isotope ratio mass spectrometry at the University Museum, University of Tokyo. The system comprised a FLASH 2000 elemental analyzer coupled to a DELTA V Advantage isotope‐ratio mass spectrometer via a ConFlo IV interface (Thermo Fisher Scientific, Waltham, MA). The isotope data (Table S2) were normalized to the international reference scales, namely, the Vienna Pee Dee Belemnite for carbon and atmospheric N_2_ for nitrogen, using internal working standards referenced to these scales (i.e., l‐alanine [AZ100 SS09, *δ* ^13^C = −19.6‰, *δ* ^15^N = 8.7‰], l‐histidine [AZ1Z0 M6M9675, *δ* ^13^C = −11.4‰, *δ* ^15^N = −7.6‰], and glycine [AZ300 M9R2283, *δ* ^13^C = −32.3‰, *δ* ^15^N = 1.12‰]; Shoko Science, Yokohama, Japan). Replicate analyses of these standards conducted at the start and after every six samples showed measurement errors (1*σ*) of ±0.1‰ for both carbon and nitrogen.

#### 2.3.2 Food Samples

All food samples were freeze‐dried and ground into fine powder using a freezer mill or mortar and pestle. The animal‐derived samples were defatted by two 30‐min ultrasonic washes in methanol–chloroform (1:2, v/v), each followed by a 5‐min centrifugation at 1500 rpm; the precipitate was rinsed with acetone in an ultrasonic bath for 10 min and then oven‐dried at 50°C. Approximately 0.2–0.4 mg of each sample were weighed into tin capsules for analysis. For non‐animal items (i.e., plant foods and most packaged convenience foods), the samples were weighed directly after freeze‐drying and grinding. In cases of low nitrogen content, samples of up to 8.0 mg were used to ensure sufficient nitrogen (≥ 20 μg, preferably 30 μg) for simultaneous carbon and nitrogen isotope analysis.

The carbon and nitrogen isotope analysis of food samples was performed using a vario PYRO cube elemental analyzer coupled to an isoprime visION IRMS (Elementar, Langenselbold, Germany) at the University Museum, University of Tokyo. The reproducibility of the measurements (1*σ*), determined from replicate analyses of the working standards (i.e., l‐alanine, l‐histidine, and glycine; Shoko Science), was less than 0.1‰ for both carbon and nitrogen.

Unless otherwise specified, the same instruments and reagents presented in Section 2.3.1 were used for the analyses.

### 2.4 Data Analysis

All analyses were performed using R version 4.5.0 (R Core Team 2025). Kernel density estimation was conducted using the rKIN package (Eckrich et al. 2020), applying smallSamp = TRUE for groups with 5 ≤ *n* < 10. Mixed‐effects models were fitted using the lme4 package (Bates et al. 2026), with *p* values obtained using lmerTest (Kuznetsova et al. 2017) and marginal and conditional *R* ^2^ calculated using performance (Lüdecke et al. 2021). The analysis code is available in the public repository listed in the Data Availability Statement. All statistical tests were two‐tailed with *α* = 0.05; the specific tests used are detailed in the table footnotes. Additionally, given the exploratory nature and limited sample sizes of the regression analyses, we report effect estimates (*b*), 95% confidence intervals, and *p* values for all variables and interpret associations with 0.05 < *p* < 0.10 cautiously, with emphasis on effect size and estimation precision rather than dichotomous significance thresholds.

To investigate the drivers of hair isotopic variation, separate multiple linear regressions were conducted for each village. This approach was based on the assumption that each village, with its distinct subsistence regime and resource use, is characterized by a different isotopic baseline and that the effects of subsistence transition and market integration on isotope values may vary across villages. For Na Savang, where most participants were sampled twice, we applied mixed‐effects models with the *individual* as a random intercept.

For each population, we constructed two sets of models to test the influence of (i) livelihood factors and (ii) dietary patterns on hair isotope values (hereinafter, the “livelihood model” [Model 1] and “dietary model” [Model 2]). This approach was based on the hypothesis that livelihood practices primarily affect hair isotope values through their influence on diet, as “diet reflects the interaction between demography, economy, environment, and food‐production technology” (O'Connell and Hedges 1999, 409). Model 1 included sociodemographic, subsistence, and economic‐related variables, whereas Model 2 included variables derived from food frequency data. Additionally, as the livelihood activities of the study populations varied with season, we accounted for seasonality in the Na Savang population by (i) including *season* as an explanatory variable in Model 1 and (ii) conducting season‐stratified analyses. All models were adjusted for age to account for potential confounding due to age‐related dietary variation. The variables included in each model are summarized in Figure S1.

Model 1 focused on variables representing the ongoing processes of subsistence transition and market integration. In Nam Nyon, paddy and swidden farming coexist and are closely linked to household economic activities and market economy participation. Here, we categorized household subsistence portfolios using hierarchical cluster analysis (Ward 1963) based on farmland area (ha; swiddens and paddy fields) and cash income (LAK; from cash crops, NTFPs, and livestock). These data were obtained from and followed the analytical method of Kibe et al. (2022). The resulting clusters were used as the “subsistence or economic variable” in Model 1. For Na Savang, where traditional subsistence activities are relatively homogeneous and dominated by wet rice cultivation, the following variables were included in Model 1 as indicators of market economy participation: *household landholding*, *occupation*, *rice self‐sufficiency*, *roofing material*, and a “possession index.” Landholding reflects productive assets and opportunities to generate cash income through crop sales. Similarly, individuals who both produced and sold rice (i.e., the “surplus” group) would have more sources of income than the “self‐sufficient” individuals. Roofing material and the possession index have previously been utilized as indicators of modernization and market integration (Mizuno et al. 2022). Concrete roofing reflects sustained financial resources and deeper market integration, as the construction usually requires several years and costs more than USD 1000. By contrast, the possession index is calculated as the sum of seven durable goods, namely, mobile phone, television, refrigerator, tractor, motorcycle, car, and truck (owned = 1, not owned = 0). The possession index is more sensitive to short‐term cash flow and represents a lower threshold of wealth accumulation. Together, these asset‐ and income‐related variables capture market integration across different time scales. As the ethnic groups in the study area may differ in resource use and dietary customs, besides the subsistence or economic variables, we included *ethnicity* in Model 1 as a sociodemographic factor to account for cultural effects not captured by the subsistence and economic variables.

To account for skewed distributions in consumption frequency data, we grouped food items with similar isotopic characteristics into broader categories and used the maximum number of intake days as explanatory variables for Model 2 (see Table S3 for the data). *Rice* and *corn* were excluded from the regression analyses due to low variability. Additionally, *corn* could not be merged with other categories as a C_4_ source. *Spices* were also excluded because of their isotopical heterogeneity (e.g., C_3_ chili pepper and C_4_ lemongrass). *Oil/fat* was retained but coded as a binary variable (days of intake ≥ 6 or not) on account of the ceiling effect in its distribution. The final food categories included in the models are presented in Figure S1.

Given the absence of data on intake amounts of individual food items and the overwhelming dominance of rice in the study populations' diet, we calculated isotopic differences between rice and hair keratin (denoted as ∆^13^C_rice–hair_ and ∆^15^N_rice–hair_). We further compared them with the accepted diet–keratin discrimination factors (O'Connell and Hedges 1999; O'Connell et al. 2012; cf. Bird et al. 2021). This enabled the qualitative assessment of the dietary contributions of rice and other foods.^2^ For each participant, ∆^13^C_rice–hair_ and ∆^15^N_rice–hair_ were determined as the difference between the isotope values of a participant's hair sample and those of rice from their corresponding (sub)population (${∆}_{\text{rice}-\text{hair}}=\delta_{\text{hair keratin}}-\delta_{\text{rice}}$). The *δ* ^13^C and *δ* ^15^N values of paddy rice from Na Savang (FSNS010) and Nam Nyon (FSNN037) were used as the subtrahend (*δ*_rice_) for the entire Na Savang population and the Nam Nyon subgroup primarily engaged in paddy field cultivation (subsistence Cluster 3; the cluster derivation is detailed in Section 3.1.2 and illustrated in Figure S2), respectively. Meanwhile, the *δ* ^13^C and *δ* ^15^N values of the upland rice sample from Nam Nyon (FSNN039) were used as the subtrahend for the Nam Nyon subgroups that mainly relied on swidden cultivation (Clusters 1 and 2).

## 3 Results

### 3.1 Isotopic Composition of Scalp Hair

A total of 175 male hair samples were analyzed from Na Savang (rainy season, *n* = 64; dry season, *n* = 59) and Nam Nyon (dry season, *n* = 52). All samples had C/N atomic ratios of 3.4–3.7 (Table S2), which is within the expected range for human keratin (2.9–3.8; O'Connell and Hedges 1999), and were retained for analysis.

Table 1 presents the descriptive statistics for hair *δ* ^13^C and *δ* ^15^N by group. *δ* ^13^C and *δ* ^15^N were negatively correlated in Nam Nyon (Pearson's *r* = −0.67, *p* < 0.001). A similar but weaker negative correlation was observed in Na Savang when both seasons were pooled (*r* = −0.21, *p* = 0.021; *n* = 123), but not within seasonal subgroups (Table S4 and Figure S3).

**TABLE 1:** Socioeconomic and isotopic characteristics of the study populations by village and sampling season.

	Na Savang [Rainy]	Na Savang [Dry]	Nam Nyon [Dry]	*p* ^a^
*N*	64	59	52	
Hair *δ* ^13^C (‰)^b^	−22.5 ± 0.5	−22.6 ± 0.6	−22.6 ± 0.4	0.93
Hair *δ* ^15^N (‰)^b^	10.8 ± 0.5	10.7 ± 0.4	7.9 ± 0.9	< 0.001
Age (years)^b^	43 ± 10	44 ± 10	35 ± 12	< 0.001
Ethnicity^c^				< 0.001
Kongsat	0 (0)	0 (0)	23 (44)	
Phunyot	0 (0)	0 (0)	27 (52)	
Yang	51 (80)	49 (83)	0 (0)	
Other	13 (20)	10 (17)	2 (3.8)	
Landholding (ha)^b^	2.1 ± 1.4	1.9 ± 1.2	2.9 ± 2.7	0.027
*Missing*	2	1	0	
Occupation^c^				0.002
Only farming	41 (64)	47 (80)	52 (100)	
Only non‐farming	2 (3.1)	0 (0)	0 (0)	
Both	21 (33)	12 (20)	0 (0)	
Rice self‐sufficiency^c^				0.004
Self‐sufficient	24 (38)	26 (44)	38 (73)	
Surplus	40 (63)	33 (56)	14 (27)	

^a^ Difference between *Na Savang [Dry]* and *Nam Nyon [Dry]*.

^b^ Mean ± standard deviation; Welch's *t*‐test.

^c^ *n* (%); Pearson's chi‐squared test.

#### 3.1.1 Interpopulation Comparison

The comparative analysis of sociodemographic and economic characteristics in the dry season (Table 1) showed that Nam Nyon participants were younger (*p* < 0.001), cultivated larger farmland areas (*p* = 0.027), and were less engaged in non‐farming occupations (*p* < 0.001) than those in Na Savang. Additionally, fewer households in Nam Nyon had surplus rice production (*p* = 0.002) than those in Na Savang. As noted in Section 2.1, ethnic composition also differed.

**FIGURE 2:**
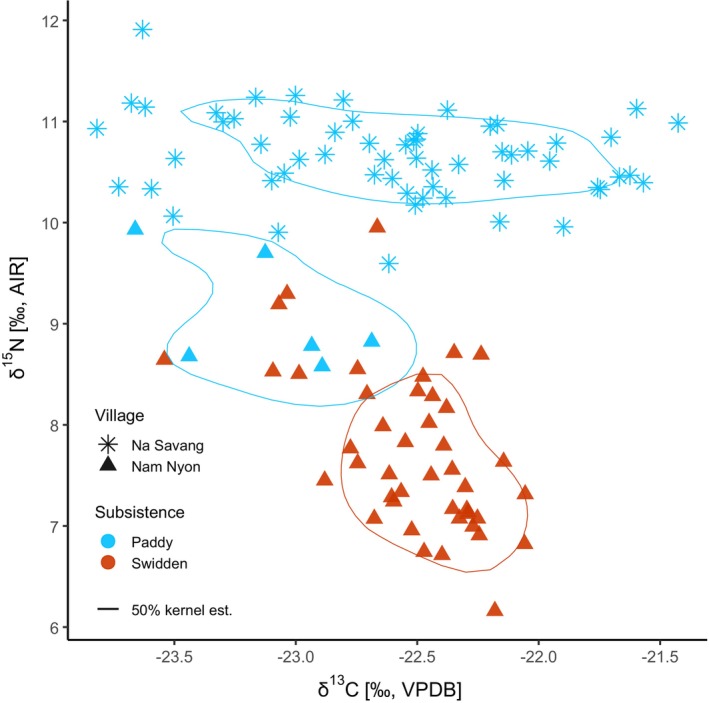
Hair *δ* ^13^C and *δ* ^15^N values (‰) of male individuals from Na Savang and Nam Nyon sampled in March 2019 (*n* = 109). The “subsistence” categories distinguish between households primarily engaged in paddy field cultivation and swidden farming. The contours indicate the 50% kernel density estimates (Eckrich et al. 2020). International reference scales: VPDB = Vienna Pee Dee Belemnite; AIR = atmospheric N_2_.

There was a clear distinction in hair isotopic composition between the villages during the dry season (Figure 2). Na Savang showed a significantly higher mean *δ* ^15^N (10.7‰ ± 0.4‰) than Nam Nyon (7.9‰ ± 0.9‰; *p* < 0.001); conversely, their mean *δ* ^13^C results were similar (Na Savang: −22.6‰ ± 0.6‰; Nam Nyon: −22.6‰ ± 0.4‰; *p* = 0.93).

#### 3.1.2 Intrapopulation Variation in Nam Nyon

Cluster analysis of Nam Nyon household subsistence portfolios revealed three distinct groups (Figure S2; see Table S5 for descriptive statistics). Cluster 1 (*n* = 17) cultivated the largest area of swiddens and earned the most from selling NTFPs in the previous year. Cluster 2 (*n* = 18) also relied on swidden farming but had minimal cash crop income. By contrast, Cluster 3 (*n* = 7) had the largest paddy fields, smallest swidden areas, highest income from livestock, and lowest income from NTFPs. Overall, Clusters 1 and 2 reflected a more “traditional” pattern of upland economy, characterized by swidden farming and NTFP trade, whereas Cluster 3 represented a more intensive, paddy‐focused livelihood.

These subsistence clusters were strongly associated with hair isotopic composition. Cluster 3 had significantly lower *δ* ^13^C and higher *δ* ^15^N than the other clusters; these differences remained significant after accounting for age and ethnicity (Table 2). Additionally, while the post hoc tests showed no direct distinction between Clusters 1 and 2 (Table S5), the regression model indicated lower *δ* ^15^N in Cluster 2 than in Cluster 1 (*b* = −0.50 [−0.95, −0.05], *p* = 0.03).

**TABLE 2:** Associations of hair isotopic compositions with livelihood‐related variables (Model 1) and dietary variables (Model 2) in Nam Nyon: Results of multiple linear regression analyses, adjusted for age.

		Hair *δ* ^13^C (‰)						Hair *δ* ^15^N (‰)					
		Model 1			Model 2			Model 1			Model 2		
Variables		*b*	(95% CI)	*p*	*b*	(95% CI)	*p*	*b*	(95% CI)	*p*	*b*	(95% CI)	*p*
Ethnicity	Phunyot (ref.)												
	Kongsat	−0.10	(−0.30, 0.10)	0.30				0.29	(−0.15, 0.73)	0.19			
	Other	−0.65	(−1.3, −0.01)	0.05				1.6	(0.14, 3.0)	0.03			
Subsistence cluster	1 (ref.)												
	2	0.05	(−0.15, 0.25)	0.62				−0.50	(−0.95, −0.05)	0.03			
	3	−0.54	(−0.85, −0.23)	< 0.01				0.92	(0.24, 1.6)	< 0.01			
Food consumption frequency													
Wild terrestrial plant					−0.03	(−0.12, 0.07)	0.57				−0.16	(−0.39, 0.06)	0.15
Riverweed/algae					−0.03	(−0.09, 0.03)	0.38				−0.01	(−0.16, 0.14)	0.93
Cultivated C_3_ plant					0.11	(0.05, 0.16)	< 0.01				−0.08	(−0.21, 0.05)	0.23
Local terrestrial animal					0.05	(−0.07, 0.17)	0.42				−0.10	(−0.41, 0.21)	0.51
Local aquatic animal					−0.07	(−0.15, 0.01)	0.07				0.13	(−0.06, 0.33)	0.17
Alcohol					0.07	(−0.01, 0.14)	0.07				0.02	(−0.17, 0.20)	0.84
Oil/fat ≥ 6 days	No (ref.)												
	Yes				−0.13	(−0.37, 0.11)	0.28				0.25	(−0.34, 0.84)	0.40
Other commercial food					−0.04	(−0.14, 0.06)	0.40				0.12	(−0.13, 0.36)	0.33
Adjusted *R* ^2^				0.31			0.24			0.40			0.13

*Note:* Model 1: *N* = 50 (2 deleted due to missingness). Model 2: *N* = 52. *b* = unstandardized regression coefficient; CI = confidence interval; ref. = reference. The subsistence clusters were identified using a hierarchical cluster analysis based on farmland area (swidden and paddy fields) and cash income. Clusters 1 and 2: swidden‐based traditional upland livelihoods; Cluster 3: paddy‐based and more market‐integrated livelihoods.

Hair *δ* ^13^C was positively associated with the consumption frequency of cultivated C_3_ plants (*b* = 0.11 [0.05, 0.16], *p* < 0.01). Meanwhile, *δ* ^15^N had no clear dietary determinants.

Overall, the livelihood models demonstrated greater explanatory power than the dietary models (adjusted *R* ^2^: 0.31 vs. 0.24 for *δ* ^13^C, 0.40 vs. 0.13 for *δ* ^15^N).

#### 3.1.3 Intrapopulation Variation in Na Savang

*Determinants of isotopic variation*: Table 3 presents the multilevel analysis results for Na Savang. Exclusive non‐farming employment was associated with higher hair *δ* ^13^C compared with exclusive farming (*b* = 0.65 [0.07, 1.2], *p* = 0.03), but this was based on only two individuals in the former category (Table 1). Household landholding (*b* = 0.05 [0.00, 0.10], *p* = 0.07) and surplus rice production (vs. self‐sufficient; *b* = 0.14 [−0.01, 0.29], *p* = 0.06) showed weak positive associations with *δ* ^13^C. Among the dietary variables, cultivated C_3_ plant consumption frequency was the only significant indicator of *δ* ^13^C (*b* = −0.04 [−0.07, −0.01], *p* = 0.02), exhibiting a negative relationship. However, the tested variables showed no clear associations with *δ* ^15^N.

**TABLE 3:** Associations of hair isotopic compositions with livelihood‐related variables (Model 1) and dietary variables (Model 2) in Na Savang: Results of mixed‐effects linear regression analyses, adjusted for age.

Fixed effects		Hair *δ* ^13^C (‰)						Hair *δ* ^15^N (‰)					
		Model 1			Model 2			Model 1			Model 2		
		*b*	(95% CI)	*p*	*b*	(95% CI)	*p*	*b*	(95% CI)	*p*	*b*	(95% CI)	*p*
Ethnicity	Yang (ref.)												
	Other	0.21	(−0.07, 0.49)	0.13				0.02	(−0.23, 0.27)	0.87			
Landholding (ha)		0.05	(0.00, 0.10)	0.07				0.00	(−0.05, 0.05)	1.00			
Occupation	Only farming (ref.)												
	Only non‐farming	0.65	(0.07, 1.2)	0.03				0.33	(−0.22, 0.87)	0.24			
	Both	−0.02	(−0.18, 0.14)	0.80				0.12	(−0.04, 0.28)	0.14			
Rice self‐sufficiency	Self‐sufficient (ref.)												
	Surplus	0.14	(−0.01, 0.29)	0.06				0.02	(−0.12, 0.17)	0.74			
Concrete roof	No (ref.)												
	Yes	0.14	(−0.06, 0.35)	0.17				−0.10	(−0.29, 0.08)	0.26			
Possession index		0.02	(−0.08, 0.13)	0.67				0.04	(−0.06, 0.13)	0.46			
Season	Dry (ref.)												
	Rainy	0.04	(−0.06, 0.15)	0.43				0.06	(−0.05, 0.16)	0.27			
Food consumption frequency													
Wild terrestrial plant					−0.01	(−0.07, 0.06)	0.88				−0.02	(−0.09, 0.04)	0.45
Riverweed/algae					0.00	(−0.03, 0.03)	0.94				−0.01	(−0.04, 0.02)	0.48
Cultivated C_3_ plant					−0.04	(−0.07, −0.01)	0.02				−0.02	(−0.05, 0.01)	0.23
Local terrestrial animal					0.00	(−0.05, 0.05)	0.90				0.00	(−0.05, 0.05)	0.94
Local aquatic animal					−0.01	(−0.06, 0.03)	0.50				0.01	(−0.03, 0.06)	0.52
Alcohol					0.01	(−0.04, 0.05)	0.83				−0.01	(−0.06, 0.03)	0.59
Oil/fat ≥ 6 days	No (ref.)												
	Yes				0.12	(−0.04, 0.28)	0.15				0.04	(−0.11, 0.20)	0.59
Other commercial food					−0.01	(−0.05, 0.04)	0.80				0.00	(−0.05, 0.04)	0.92

		Var.	ICC		Var.	ICC		Var.	ICC		Var.	ICC	
Random effects													
Individual	(Intercept)	0.20	0.76		0.24	0.79		0.13	0.67		0.12	0.64	
Residual		0.06			0.06			0.06			0.07		
Model fit													
Conditional *R* ^2^				0.80			0.81			0.68			0.65
Marginal *R* ^2^				0.17			0.10			0.05			0.03

*Note:* Model 1: *N* of observations = 120; *N* of groups (individual) = 70 (3 observations from 2 individuals deleted due to missingness). Model 2: *N* of observations = 122; *N* of groups = 72 (1 observation from 1 individual deleted due to missingness). *b* = unstandardized regression coefficient; CI = confidence interval; ref. = reference; Var. = variance; ICC = intraclass correlation coefficient.

As in Nam Nyon, the livelihood models were generally more explanatory than the dietary models (marginal *R* ^2^: 0.17 vs. 0.11 for *δ* ^13^C, 0.06 vs. 0.03 for *δ* ^15^N). Nevertheless, the consistently high intraclass correlation coefficient (ICC) values and large discrepancies between marginal and conditional *R* ^2^ indicate substantial stable heterogeneity across individuals not captured by the observed fixed effects.

*Seasonal comparison*: While paired *t*‐tests suggested no seasonal differences in mean *δ* ^13^C or *δ* ^15^N (*p* > 0.05; Table S6), the socioeconomic indicators showed season‐specific patterns in their associations with *δ* ^13^C (Table S7). In the rainy season, surplus rice production (*b* = 0.22 [−0.04, 0.48], *p* = 0.09) and the possession index (*b* = 0.12 [−0.02, 0.26], *p* = 0.09) showed positive but weak associations with hair *δ* ^13^C. In the dry season, participants from “other” ethnic groups had higher hair *δ* ^13^C than the dominant Yang (*b* = 0.46 [0.04, 0.89], *p* = 0.03), and surplus rice production was also positively associated with *δ* ^13^C (*b* = 0.31 [0.01, 0.61], *p* = 0.04). Landholding (*b* = 0.11 [−0.01, 0.23], *p* = 0.07) and concrete roofing (*b* = 0.28 [−0.03, 0.59], *p* = 0.08) showed additional positive associations, although the estimates were less precise.

### 3.2 Isotopic Baseline of Foodstuffs

**FIGURE 3:**
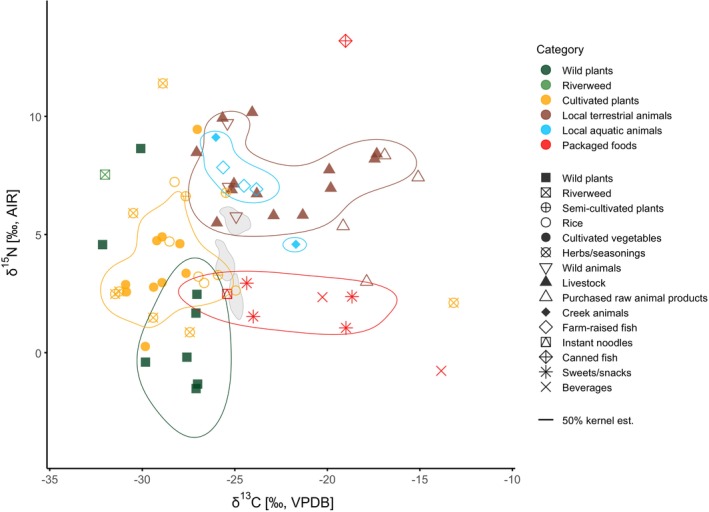
*δ* ^13^C and *δ* ^15^N values (‰) of food samples collected in the study area (*n* = 67). The contours indicate the 50% kernel density estimates (Eckrich et al. 2020). The gray‐shaded areas show the 50% kernel density estimates for scalp hair samples grouped as in Figure 2 and corrected for diet–keratin discrimination factors (+2.5‰ for carbon and +5.15‰ for nitrogen; O'Connell and Hedges 1999; O'Connell et al. 2012; cf. Bird et al. 2021). International reference scales: VPDB = Vienna Pee Dee Belemnite; AIR = atmospheric N_2_.

Table 4 and Figure 3 present the carbon and nitrogen isotopic compositions of local foods (see Table S1 for raw data). The plant samples were predominantly C_3_, with lemongrass ( *Cymbopogon citratus* ) being the only C_4_ species. The mean *δ* ^13^C was −28.5‰ ± 2.0‰ for wild plants and −27.9‰ ± 3.5‰ for cultivated plants. Moreover, *δ* ^15^N values differed between wild and cultivated plants, with means of 1.7‰ ± 3.5‰ and 4.1‰ ± 2.6‰, respectively. Riverweed (*Cladophora* spp.), the only aquatic plant sampled, had lower *δ* ^13^C and higher *δ* ^15^N than terrestrial plants.

**TABLE 4:** Isotopic characteristics of food samples collected in the study area by category.

Category	*δ* ^13^C (‰)			*δ* ^15^N (‰)		
	*N*	Mean	SD	*N*	Mean	SD
Wild plants	8	−28.5	2.0	8	1.7	3.5
Riverweed	1	−31.9	—	1	7.6	—
Cultivated plants	25	−27.9	3.5	25	4.1	2.6
Semi‐cultivated plants	3	−26.4	1.2	3	5.6	1.9
Rice	5	−27.1	1.5	5	4.2	1.9
Cultivated vegetables	10	−29.1	1.3	10	3.9	2.4
Herbs/seasonings	7	−27.4	6.5	7	3.9	3.7
Local terrestrial animals	20	−22.0	3.7	20	7.2	1.8
Wild animals	3	−25.3	0.2	3	7.4	2.0
Livestock	13	−22.7	3.3	13	7.5	1.5
Purchased raw animal products^a^	4	−17.3	1.7	4	6.1	2.4
Local aquatic animals	5	−24.3	1.7	5	7.1	1.6
Creek animals	2	−23.9	3.1	2	6.8	3.2
Farm‐raised fish	3	−24.6	1.0	3	7.3	0.5
Packaged foods	10	−19.7	4.5	8	3.1	4.2
Instant noodles	1	−25.4	—	1	2.6	—
Canned fish	1	−18.9	—	1	13.2	—
Sweets/snacks	5	−19.5	5.2	4	2.0	0.8
Beverages	3	−18.5	4.1	2	0.7	2.2

Abbreviation: SD = standard deviation.

^a^ Includes raw meat and eggs.

Special attention was paid to rice, the staple food and principal energy source for the study populations. Among the five samples, paddy rice had lower *δ* ^13^C and higher *δ* ^15^N than upland rice (Figure S4). For paddy rice, the Na Savang sample was 2.4‰ higher in *δ* ^15^N than the Nam Nyon sample (Table S1).

Meat from three wild terrestrial animals with different ecological niches—namely, bird (Aves), squirrel (Sciuridae), and barking deer (*Muntiacus* spp.)—had a mean *δ* ^13^C of −25.3‰ ± 0.2‰. Livestock generally showed higher *δ* ^13^C but varied by source. Those from markets (mostly Na Savang) and those sampled in the Akha villages generally had higher *δ* ^13^C than wild animals, whereas livestock from Nam Nyon were similar to wild animals (Figure S5). Aquatic animals fell within the ranges observed for terrestrial animals for *δ* ^13^C and *δ* ^15^N (Figure 3) and were thus not separable on either axis.

Most market‐derived packaged foods showed *δ* ^13^C similar to terrestrial animals but much lower *δ* ^15^N (means: −19.7‰ ± 4.5‰ vs. −22.0‰ ± 3.7‰ for *δ* ^13^C; 3.1‰ ± 4.2‰ vs. 7.2‰ ± 1.8‰ for *δ* ^15^N), with the exception of canned mackerel, which was enriched in both isotopes. Two market food samples lacked valid *δ* ^15^N measurements due to insufficient nitrogen content but had relatively high *δ* ^13^C values (−21.4‰ and −11.6‰; Table S1).

### 3.3 Rice–Hair Isotopic Offset

The mean ∆^13^C_rice–hair_ and ∆^15^N_rice–hair_ values exhibited a reverse relationship across the (sub)populations (Table 5). Nam Nyon individuals in Cluster 1 had the lowest ∆^13^C_rice–hair_ (+2.4‰ ± 0.3‰) and highest ∆^15^N_rice–hair_ (+5.3‰ ± 0.8‰), closely followed by those in Cluster 2 (+2.5‰ ± 0.4‰ for carbon, +4.8‰ ± 0.8‰ for nitrogen). These values align with the diet–keratin discrimination factors of approximately +2.5‰ for carbon and +5.0‰–5.3‰ for nitrogen suggested by previous investigations (O'Connell et al. 2012; O'Connell and Hedges 1999) and applied in global‐scale dietary isotope comparisons (Bird et al. 2021). Conversely, higher ∆^13^C_rice–hair_ and lower ∆^15^N_rice–hair_ relative to the accepted diet–keratin offsets were observed for Nam Nyon villagers in Cluster 3 (+5.4‰ ± 0.4‰ for carbon, +4.3‰ ± 0.6‰ for nitrogen) and for Na Savang residents (+5.7‰–5.8‰ for carbon, +3.5‰–3.6‰ for nitrogen).

**TABLE 5:** Rice–hair isotopic offsets of the study populations by village, sampling season, and subsistence cluster (identified by hierarchical cluster analysis).

	*N*	∆^13^C_rice–hair_ (‰)	∆^15^N_rice–hair_ (‰)
Na Savang			
Rainy season	64	5.8 ± 0.5	3.6 ± 0.5
Dry season	59	5.7 ± 0.6	3.5 ± 0.4
Nam Nyon			
Cluster 1	24	2.4 ± 0.3	5.3 ± 0.8
Cluster 2	20	2.5 ± 0.4	4.8 ± 0.8
Cluster 3	6	5.4 ± 0.4	4.3 ± 0.6

*Note:* The data are shown as mean ± standard deviation. The subsistence clusters were identified using a hierarchical cluster analysis based on farmland area (swidden and paddy fields) and cash income. Clusters 1 and 2: swidden‐based traditional upland livelihoods; Cluster 3: paddy‐based and more market‐integrated livelihoods.

## 4 Discussion

### 4.1 Food Isotopic Baseline in Northern Laos

The isotopic landscape of local foods was dominated by C_3_ resources. Wild and (semi‐)cultivated plants had similar *δ* ^13^C but differed in *δ* ^15^N. The tendency of (semi‐)cultivated plants to have higher *δ* ^15^N than wild plants likely reflects the effects of human activities on local nitrogen cycles. Even without deliberate manuring, soils around villages can be enriched in ^15^N, possibly due to inputs from human and livestock waste (Commisso and Nelson 2010; Dupouey et al. 2002; Lake et al. 2001). Therefore, plants growing in these areas (e.g., semi‐cultivated plants and cultivated vegetables or seasoning plants) are expected to have elevated *δ* ^15^N values. Moreover, although swiddens are usually located at some distance from human settlements, burning vegetation can lead to an increase in soil and plant *δ* ^15^N (Högberg 1997; Huber et al. 2013; Saito et al. 2007). This could be because isotopic fractionation during combustion favors the gaseous loss of the lighter ^14^N (Turekian et al. 1998; cf. Szpak 2014). In this study, two wild plant samples had unusually high *δ* ^15^N, namely, *Gnaphalium* spp. (4.5‰) and Phyllanthaceae (8.7‰), likely due to local microenvironments such as those with proximity to farmland, animal excrement sites, or carcasses.

Paddy rice was depleted in ^13^C and enriched in ^15^N compared with upland rice (Figure S4). This finding was expected because, unlike paddy rice, which grows in water‐rich environments, upland rice typically suffers from water stress. This prompts the stomata on C_3_ plant leaves to partially close to reduce water loss, diminishing intercellular CO_2_ concentration and increasing ^13^C uptake (Farquhar et al. 1982). Higher *δ* ^15^N in paddy rice can be attributed to proximity to villages and the dominant process of denitrification in water‐saturated soils, which preferentially removes the lighter ^14^N and leaves a ^15^N‐enriched soil nitrate pool for plant uptake (Choi et al. 2003; Ishii et al. 2011; Mariotti et al. 1981). Experimental (Yoneyama et al. 1990) and archaeological research (Shoda et al. 2021) further confirms the higher *δ* ^15^N in paddy rice relative to upland crops without fertilizer use.

Notably, the paddy rice sample from Na Savang had considerably higher *δ* ^15^N than that from Nam Nyon, whereas their *δ* ^13^C values were similar (Figure S4 and Table S1). Very high *δ* ^15^N values in rice have been reported previously, including in commercially purchased rice from an inland port city in East Africa (Correia et al. 2019). The *δ* ^15^N value observed in Na Savang (7.2‰) nevertheless exceeded almost all rice samples reported by Korenaga et al. (2010), who analyzed 163 samples from six countries, mostly cultivated with artificial fertilizer or natural manure. This high *δ* ^15^N can be attributed to the long history of paddy rice cultivation in Na Savang, where over 69% of the paddies were developed before 1960 (cf. Tomita 2008, 2020). By contrast, Nam Nyon only adopted paddy fields in the 1980s, when the Kongsat first moved to their current location. Soil organic *δ* ^15^N in open systems rises with soil age (Martinelli et al. 1999), whereas paddy soil *δ* ^15^N with manure composts increases with the duration of cultivation (Nishida et al. 2007). In the study villages, paddy fields were also exposed to livestock manure, whether applied manually or deposited naturally by the animals. Therefore, it is possible that the variation in *δ* ^15^N between paddy soils with differing cultivation ages resulted in the variation in *δ* ^15^N among the paddy rice samples.

Wild terrestrial animals with varying predatory habits (i.e., the bird, squirrel, and barking deer) clustered at *δ* ^13^C ≈ −25.3‰ with minimal dispersion. Considering the effect of defatting for muscle (~+0.5‰; cf. Cloyed et al. 2020) and a diet–consumer offset of +0.9‰–3.0‰ for C_3_‐fed birds and mammals (∆^13^C_diet–muscle_; cf. R. B. Stephens et al. 2023), wild animals' *δ* ^13^C aligned well with those of wild plants (−28.5‰ ± 2.0‰; Table 4 and Figure 3). Therefore, this study's wild plant and animal samples provide a practical carbon isotope baseline for the terrestrial food web supporting local human populations, albeit one that should be regarded as provisional given the modest number of samples analyzed.

Furthermore, all domesticated animal samples with higher *δ* ^13^C signals were from Na Savang (market‐sourced) or the Akha villages, whereas samples from Nam Nyon resembled the wild animal baseline (Figure S5). This difference reflects feeding patterns, with maize (C_4_) commonly grown and used as fodder in the Akha villages and Na Savang but not in Nam Nyon.

Indistinguishable isotopic compositions of freshwater and terrestrial animals were consistent with the patterns found in archaeological studies (Dufour et al. 1999; Hedges and Reynard 2007). This limited differentiation is attributable to the substantial variability of freshwater resources in both *δ* ^13^C and *δ* ^15^N. Such variability arises from variations in habitat baselines and trophic structure within freshwater ecosystems, which can cause freshwater fauna to overlap isotopically with C_3_‐based terrestrial herbivores and carnivores (Dufour et al. 1999; Guiry 2019; Marchenko et al. 2021; Syväranta et al. 2006).

The low *δ* ^15^N values of most convenience foods indicate their plant origins. Their elevated *δ* ^13^C values likely reflect the use of C_4_‐derived sweeteners, particularly cane sugar and high‐fructose corn syrup, which are common ingredients in processed foods and beverages (Jahren et al. 2006). These results suggest the incorporation of industrial C_4_ carbon into the local human food web.

### 4.2 Interpopulation Divergence and Dietary Implications

It is difficult to determine from the study data whether the higher hair *δ* ^15^N in Na Savang compared with Nam Nyon reflects only the elevated *δ* ^15^N of rice or the greater consumption of animal‐derived protein as well. Participants from Na Savang reported more frequent consumption of terrestrial animal meats and products (Table S3). Alternatively, it is possible that the higher frequency of wild plant consumption among Nam Nyon residents contributed to the difference in hair *δ* ^15^N between the two villages.

The isotopic differences between hair keratin and rice provide further insights. For Nam Nyon villagers with traditional upland subsistence portfolios (Clusters 1 and 2), the consistency between their ∆^13^C_rice–hair_ and ∆^15^N_rice–hair_ and the reference diet–keratin offsets indicate that the isotope ratios of upland rice are reliable proxies of their dietary isotope values. Conversely, individuals relying on paddy field cultivation (Cluster 3 of Nam Nyon and all participants from Na Savang) exhibited higher ∆^13^C_rice–hair_ and lower ∆^15^N_rice–hair_ relative to the reference values. This suggests a diet enriched in ^13^C and depleted in ^15^N compared with paddy rice. The simplest explanation for this isotopic “gap” is the additional consumption of commercial foods, including instant noodles and sweetened snacks or beverages, which have higher *δ* ^13^C and lower *δ* ^15^N than paddy rice. An alternative source is corn, which was not sampled in this study but is expected to have high *δ* ^13^C and low *δ* ^15^N as a C_4_ crop typically grown in upland fields. However, the reported frequency of corn consumption was much lower than that of commercial foods in both villages (Tables S3 and S6).

Significantly, given this study's limited number of rice samples (*n* = 1 for each group) and the variation in reported diet–hair enrichment values (e.g., Hedges and Reynard 2007; Hedges et al. 2009; O'Connell et al. 2012; Yoshinaga et al. 1996), the foregoing interpretations of rice–hair differences should be treated with caution.

### 4.3 Intrapopulation Dynamics

We employed two multiple linear regression models to investigate the livelihood and dietary determinants of variation in hair isotopic compositions within the target populations. Models based on livelihood‐related variables explained considerably more variation than those based on dietary frequency. This difference likely reflects limitations in relating human isotopic values to questionnaire‐based dietary measures. First, questionnaire data usually provide consumption frequency rather than actual intake amounts, and are thus susceptible to ceiling effects for very frequently consumed foods (e.g., Ryman et al. 2014). In our case, the questionnaire recorded only the number of days each food item was consumed; collected data could not account for differences in the relative consumption of foods by weight, particularly the much smaller contribution of non‐rice foods compared with rice, as shown by a direct‐weighing survey in Nam Nyon (Kibe et al. 2022).^3^ Second, the temporal scale of dietary reporting may not match that represented by biological sampling (e.g., Valenzuela et al. 2018). Here, our questionnaire accounted for food consumption frequency during the previous week, whereas the 1‐cm scalp‐proximal hair segments roughly corresponded to the previous month. Finally, isotopic variation within food categories may be attenuated when foods are grouped into dietary variables (e.g., Patel et al. 2014; Valenzuela et al. 2018; cf. Huelsemann et al. 2013).

#### 4.3.1 Socioeconomic Drivers of Isotopic Variation in Nam Nyon

Among the Nam Nyon participants, the significantly higher *δ* ^15^N and lower *δ* ^13^C in the hair of paddy field cultivators (Cluster 3) than those of swidden cultivators (Clusters 1 and 2) were consistent with the difference in isotopic compositions between paddy and upland rice (Figure S4).

After accounting for age and ethnicity, individuals in Cluster 2 showed significantly lower hair *δ* ^15^N than those in Cluster 1. Although collectively interpreted as representing the traditional upland subsistence, Cluster 1 might have had a more positive engagement in the market economy, as indicated by a larger cultivation area of swiddens and higher income from cash crops (Table S5). Moreover, Cluster 2 might have consumed more ^15^N‐depleted wild plants, as inferred from their lower ∆^15^N_rice–hair_ offsets compared with Cluster 1 (Table 5).

#### 4.3.2 Socioeconomic Drivers of Isotopic Variation in Na Savang

In the main model, both an inheritable asset (i.e., household landholding) and cash‐earning activities (i.e., exclusively non‐farming employment and rice sales) showed positive associations with hair *δ* ^13^C values, although some effect sizes were small and confidence intervals overlapped zero (Model 1, Table 3). This suggests that individuals with greater economic resources in Na Savang may have consumed diets slightly enriched in ^13^C, potentially reflecting greater intake of animal‐derived protein and/or market foods.

While the mean hair *δ* ^13^C did not differ significantly between seasons, the stratified models suggested possible seasonal variation in the socioeconomic drivers of *δ* ^13^C (Table S7), aligning with the seasonal fluctuations in local agricultural labor. The month of March, during which the dry season sampling was conducted, is considered the off‐season in the traditional local farming calendar. During this period, short‐term cash flow (i.e., rice sales) and a social structural factor (i.e., ethnicity) appear to have contributed to higher dietary *δ* ^13^C; long‐term assets (i.e., household landholding and roofing material) were also associated, albeit more weakly. Meanwhile, during the labor‐intensive rainy season, people are busy with farm work, especially with weeding rice paddies. In this season, the effects of long‐term structural factors were unclear, and only two variables more responsive to short‐term cash flows and early market engagement (i.e., rice sales and the possession index) showed subtle associations with elevated *δ* ^13^C. Food frequency data are also broadly consistent with this pattern. In Na Savang, the participants reported eating market‐based foods (e.g., canned fish and oil) more often during the rainy season and a higher consumption frequency of foods obtained through hunting and gathering activities (e.g., riverweed, bushmeat, and fish organs) during the dry season (Table S6). Taken together, these results suggest that market integration may contribute to dietary *δ* ^13^C variation in two ways, namely, as a “strategic consumption” response to short‐term labor demand and a “lifestyle consumption” pattern related to long‐term wealth accumulation.

The regression models demonstrated substantially greater explanatory power for *δ* ^13^C than for *δ* ^15^N (Table 3). Although *δ* ^15^N is commonly used to indicate trophic level, this interpretation may be inappropriate given the specific agroecological context of Na Savang. This is because rice from these long‐cultivated paddies has unusually high *δ* ^15^N, comparable to that of animal‐derived foods (Figure 3). Given the dominance of rice in the subsistence‐based local diet, socioeconomic differentiation is unlikely to translate into differentiation in *δ* ^15^N signals, accounting for the low intrapopulation variability in *δ* ^15^N and the absence of clear associations in our models. By contrast, *δ* ^13^C effectively distinguishes commercial foods and corn‐fed livestock (high *δ* ^13^C) from C_3_ plants (low *δ* ^13^C), such that “non‐traditional” dietary choices (i.e., higher meat and market food consumption) are more likely to be reflected as higher *δ* ^13^C values.

It is worth reiterating that both the main and season‐stratified regression analyses are exploratory. Across these models, several estimated effects were small, with confidence intervals that included zero, and the modest sample sizes (particularly in the seasonal subsamples and sparse categories) limited the precision of the estimates. Therefore, rather than definitive evidence, the observed associations should be regarded as suggestive patterns that warrant further investigation. More broadly, the high ICC values and large discrepancies between marginal and conditional *R* ^2^ of the mixed‐effects models indicate that much of the variance in hair isotope values was attributable to individual‐level differences rather than to the examined covariates alone, implying the contribution of additional unmeasured or longer‐term factors to the observed isotopic variation.

#### 4.3.3 Dietary Determinants

The consumption frequency of cultivated C_3_ plants showed opposite effects on hair *δ* ^13^C in Na Savang and Nam Nyon. As these foods are typically ^13^C‐depleted in the study villages, the negative effect observed in Na Savang was expected (Table 3), whereas the significant positive effect (*b* = 0.11 [0.05, 0.16], *p* < 0.01; Table 2) in Nam Nyon was puzzling. Nevertheless, these two scenarios can be reconciled if cultivated C_3_ plant intake is interpreted as a proxy for relatively “traditional” subsistence patterns in each village. In Na Savang, where the traditional livelihoods are centered on paddy rice farming, greater participation in the market economy (e.g., through rice sales or exclusively non‐farm employment) may be associated with diets containing more ^13^C‐enriched foods and thus higher hair *δ* ^13^C values. By contrast, in Nam Nyon, the transition from the traditional upland subsistence based on swidden farming and NTFP trade toward a more intensive, paddy‐oriented livelihood may have resulted in a decrease in dietary and hair *δ* ^13^C, as suggested by the lower *δ* ^13^C in Cluster 3 compared to Clusters 1 and 2. Taken together, these findings illustrate how the isotope patterns associated with “traditional” subsistence depend on the specific livelihood system involved, and how market integration may influence hair *δ* ^13^C through multiple context‐specific pathways.

### 4.4 Common Patterns and Broader Implications

In the study area, paddy field ownership likely signifies greater economic opportunities and is thus linked to stronger market involvement. This finding is supported by local narratives, as most cash crops (e.g., tobacco, watermelon, and numerous vegetables) are grown in paddy fields, as well as by isotopic evidence from the higher ∆^13^C_rice–hair_ values among paddy owners.

The sampled populations had lower hair *δ* ^13^C values than those reported for many modern populations, particularly industrialized or market‐integrated groups, as reviewed in Bird et al. (2021) and Hülsemann et al. (2015). This finding can be attributed to climate factors, as northern Laos has a typical annual precipitation of 1200–1800 mm (World Bank 2025), which is a predictor of low *δ* ^13^C signatures in terrestrial C_3_ plants (Cornwell et al. 2018; Kohn 2010). Additionally, relatively low intake of C_4_‐derived sweeteners, which are commonly consumed in industrialized societies, may contribute to the low *δ* ^13^C values of the study populations. Mirroring the pattern observed in Na Savang, as the market economy permeates the region, local people's hair *δ* ^13^C is expected to increase due to the elevated consumption of C_4_‐derived foods (e.g., packaged foods and C_4_‐fed animals).

Similar isotopic variation resulting from dietary shifts during market integration has been reported by studies examining the indigenous populations of Greenland (Bjerregaard et al. 2017; Buchardt et al. 2007), Brazil (Nardoto et al. 2006, 2011, 2020), Southwest Alaska (Choy et al. 2019; Nash et al. 2012; Ryman et al. 2014), and Papua New Guinea (Umezaki et al. 2016). Although positive correlations between *δ* ^13^C and *δ* ^15^N are more commonly observed, rare negative correlations have also been found in urban and rural Brazilian communities (Nardoto et al. 2006, 2011, 2020), as well as in the present study. More generally, the exact direction of such an “isotopic shift” in each population is determined by the specific isotopic niches of local (traditional) and market‐introduced (non‐traditional) foods. The negative correlation between hair *δ* ^13^C and *δ* ^15^N observed in Na Savang may be a concrete example of this shift, with paddy rice and market‐derived foods serving as two possible endpoints of the dietary isotope spectrum. Bird et al. (2021) observed an overall reduction in isotope dietary breadth resulting from the industrialized food production and globalized food distribution. In this study, however, we observed an initial broadening of dietary breadth while introducing new isotopic niches in the early stages of market integration. Nevertheless, if local foods are progressively replaced by market‐supplied foods, dietary isotope signals in formerly self‐sufficient populations may “converge” toward the center of the *δ* ^13^C–*δ* ^15^N biplot and eventually join those of urban populations (cf. fig. 1 in Bird et al. 2021).

The findings of this study have several implications for future research. Archaeological interest has been directed toward nitrogen stable isotope in areas dominated by C_3_ plant resources (Szpak 2014). However, this study suggests that carbon isotope values may serve as a useful indicator of market integration for modern subsistence populations in these regions, where the dietary contribution of C_4_‐derived foods may be easier to detect through elevated *δ* ^13^C values against a low‐corn dietary background (O'Brien 2015). The plant isotope values reported in this study, especially those of wild plants, could serve as paleodietary baselines in similar regions, as wild species and, to a lesser extent, self‐sufficient crops, were not subjected to chemical fertilizers. Finally, this study provides new evidence that cereals and other cultivated crops exhibit systematically higher *δ* ^15^N values than wild plants and/or herbivore forage. This is an important source of bias that can lead to the overestimation of animal protein proportion in paleodietary reconstructions based on the *δ* ^15^N values of human and herbivore remains (Hedges and Reynard 2007), alongside the underestimated ∆^15^N_diet–collagen_ offset discussed by O'Connell et al. (2012). The findings of this study also indicate that the *δ* ^15^N difference between cultivated and wild plants can be as large as several trophic levels, especially in sedentary agricultural societies.

### 4.5 Strengths and Limitations

This study is the first to report on the natural abundances of carbon and nitrogen stable isotopes in Lao PDR and among the few to focus on modern subsistence populations. This focus is especially relevant given that foraging and subsistence‐oriented lifeways are rapidly changing under increasing market integration. Moreover, two of the authors (S.T. and M.K.) have extensive anthropological field experience in the study villages, with S.T. having conducted fieldwork in Na Savang over a span of 20 years and M.K. in Nam Nyon for 13 months. This experience helped ground our interpretations in a deep understanding of human–environment interactions within local ecosystems.

However, this study has some limitations. Direct comparisons between the two study populations are constrained by differences in seasonal sampling and by the use of population‐specific subsistence and economic variables. Additionally, the modest sample sizes reduced the statistical power and precision of the regression estimates. Another limitation is the small number of food samples, particularly for each species, and the inconsistent timing of food and hair collection. Although these factors may affect the applicability of the food samples as baselines for our hair analyses, they can still serve as useful isotopic references for the region.

## 5 Conclusion

This study demonstrated how subsistence practices and dietary behaviors are closely interconnected and how these interactions can be captured by stable isotope patterns. We provided concrete cases of isotopic niche differentiation resulting from subsistence transition and socioeconomic differentiation during the early stages of market integration in subsistence populations. The analysis revealed higher hair *δ* ^15^N in the lowland paddy‐farming village than in the upland swidden‐farming village, along with an increase in *δ* ^15^N and decrease in *δ* ^13^C associated with the shift from swidden to paddy farming. Furthermore, in the lowland village, individuals with greater market involvement exhibited higher hair *δ* ^13^C values. Therefore, market integration can lead to an increase in hair *δ* ^13^C through the consumption of C_4_‐derived foods, with the overall isotopic outcomes remaining highly context dependent.

## Author Contributions

**Ziyang Li:** conceptualization (lead); data curation (lead); formal analysis (lead); funding acquisition (supporting); investigation (lead); methodology (lead); validation (lead); writing – original draft (lead); writing – review and editing (lead). **Mihoko Kibe:** conceptualization (supporting); data curation (lead); funding acquisition (supporting); investigation (equal); validation (supporting); writing – review and editing (supporting). **Yuki Mizuno:** data curation (supporting); investigation (equal); methodology (supporting); writing – review and editing (supporting). **Masafumi Saitoh:** data curation (supporting); investigation (equal); writing – review and editing (supporting). **Kohei Yamazaki:** data curation (supporting); investigation (equal); writing – review and editing (supporting). **Hiroaki Masuoka:** investigation (equal); writing – review and editing (supporting). **Satoko Kosaka:** investigation (equal); writing – review and editing (supporting). **Kazumi Natsuhara:** investigation (equal); writing – review and editing (supporting). **Kazuhiro Hirayama:** investigation (equal), writing – review and editing (supporting). **Nouhak Inthavong:** data curation (lead); investigation (equal); project administration (supporting); writing – review and editing (supporting). **Sengchanh Kounnavong:** project administration (equal); writing – review and editing (supporting). **Minoru Yoneda:** funding acquisition (supporting); methodology (equal); resources (equal); writing – review and editing (supporting). **Shinsuke Tomita:** conceptualization (supporting); data curation (lead); funding acquisition (lead); investigation (equal); project administration (equal); validation (supporting); writing – review and editing (supporting). **Masahiro Umezaki:** conceptualization (lead); funding acquisition (lead); investigation (equal); project administration (equal); supervision (lead); writing – original draft (supporting); writing – review and editing (supporting).

## Funding

This work was funded by JSPS KAKENHI (Japan Society for the Promotion of Science) (Grant Numbers JP17H02233, JP19H03315, JP20K21443, JP21H03684, JP21J12835, JP25K24680) and JST SPRING (Japan Science and Technology Agency) (Grant Number JPMJSP2108).

## Ethics Statement

This study was approved by the Research Ethics Committee of the Graduate School of Medicine, The University of Tokyo [approval number: 12033‐(7)], and the National Ethics Committee for Health Research, Ministry of Health, Lao PDR (approval numbers: 2018.22.MP, 2022.26, 2024.61). Prior to data collection, meetings were held with village leaders and local health authorities to explain the objectives and procedures of the study, and written informed consent was obtained from each participant. Study findings will be shared with participating communities and local stakeholders through community meetings and summary reports prepared in the local language where feasible.

## Conflicts of Interest

The authors declare no conflicts of interest.

## Supporting information


**Figure S1:** Independent variables in the multilinear regression models for each village. The “subsistence cluster” refers to household categories obtained from a hierarchical cluster analysis based on farmland area and cash income (Figure S2). The “stock” variables represent long‐term assets, whereas the “flow” variables indicate short‐term income sources.
**Figure S2:** Clustering of 42 households in Nam Nyon by subsistence portfolios (Ward 1963), using farmland area and cash income as clustering variables (see Table S5).
**Figure S3:** Linear correlation between hair *δ* ^13^C and *δ* ^15^N values (‰) of male individuals from Na Savang and Nam Nyon sampled in August 2018 and March 2019 (*n* = 175). Regression lines are shown for each village, with the shaded areas representing 95% confidence intervals. Pearson correlation *r* = −0.21 for Na Savang (*n* = 123, *p* = 0.021) and *r* = −0.67 for Nam Nyon (*n* = 52, *p* < 0.001).
**Figure S4:** Distribution of *δ* ^13^C and *δ* ^15^N values (‰) of rice samples (*n* = 5). Sampling villages are indicated by the labeled boxes adjacent to each data point.
**Figure S5:** Distribution of *δ* ^13^C and *δ* ^15^N values (‰) of terrestrial animal samples (*n* = 20).
**Table S3:** Comparison of food consumption frequency (days of intake in the past week) between males from Na Savang and Nam Nyon surveyed in March 2019 (dry season).
**Table S4:** Linear correlation between hair *δ* ^13^C and *δ* ^15^N values (‰) of males from Na Savang and Nam Nyon sampled in August 2018 (rainy season) and March 2019 (dry season).
**Table S5:** Comparisons of household‐ and individual‐level variables among the subsistence clusters identified by the hierarchical cluster analysis in Nam Nyon (Figure S3).
**Table S6:** Comparisons of isotopic, anthropometric, socioeconomic, and food consumption frequency data for males from Na Savang who participated in both August 2018 (rainy season) and March 2019 (dry season) surveys (*n* = 51).
**Table S7:** Associations between hair *δ* ^13^C (‰) and socioeconomic variables in Na Savang, stratified by sampling season: Results of multiple linear regression analyses, adjusted for age.

**Table S1:** Detailed information and isotopic composition of food samples collected in northern Laos.
**Table S2:** Isotopic composition and related information of scalp hair samples collected in northern Laos.

## Data Availability

Stable carbon and nitrogen isotope measurements for food and scalp hair samples are available in the Supporting Information and, together with the R code supporting the analyses in this article, have been deposited in Zenodo: 10.5281/zenodo.19889732. For confidentiality reasons, anthropometric and sociodemographic data are not publicly available, as individuals could be identifiable due to the small populations of the study villages.

## Endnotes

^1^
Sampling was intended to cover the major dietary resources and/or foods considered informative for dietary isotope variation. Some questionnaire items were not analyzed because they were rarely consumed in the study villages (e.g., insects/larvae and processed meats), unavailable during the sampling period (e.g., fermented animal products and fish organs), or typically used as cooking ingredients rather than consumed as discrete food items (e.g., oil/fat).

^2^
The discrimination factors (∆) between diet and consumer, especially for nitrogen, can fluctuate with physiological status (Reitsema 2013); for instance, they can increase under protein deficiency (Naito et al. 2015) and decrease during anabolic states due to growth (Waters‐Rist and Katzenberg 2010) or pregnancy (Fuller et al. 2004). As all participants in this study were male adults and the typical local diet provided adequate protein intake (Kibe et al. 2022), interindividual variation in ∆ was assumed to be negligible.

^3^
Although Kibe et al. (2022) presented more detailed dietary data from a direct‐weighing survey in Nam Nyon, their data were not used in this study because the survey targeted only 14 of the 44 households in the village. Moreover, the survey was conducted during the rainy season (August and September 2019), whereas the hair samples analyzed in this study were collected during the dry season (March 2019). Long‐term field investigations in the study villages (Kibe et al. 2022; Tomita et al. 2015) have suggested marked seasonal variation in dietary sources, although they may not differ isotopically.
