# Supplementary material for: Livelihood and Dietary Transitions in Subsistence Populations of Northern Laos: Insights From Carbon and Nitrogen Stable Isotope Analysis
Source: Am J Biol Anthropol. 2026 Jul 31;190(4):e70326. doi: 10.1002/ajpa.70326 (PMC13428189; doi:10.1002/ajpa.70326)
Supplement: Supplementary file 1 — Figure S1: Independent variables in the multilinear regression models for each village. The “subsistence cluster” refers to household categories obtained from a hierarchical cluster analysis based on farmland area and cash income (Figure S2). The “stock” variables represent long‐term assets, whereas the “flow” variables indicate short‐term income sources. Figure S2: Clustering of 42 households in Nam Nyon by subsistence portfolios (Ward 1963), using farmland area and cash income as clustering variables (see Table S5). Figure S3: Linear correlation between hair δ 13C and δ 15N values (‰) of male individuals from Na Savang and Nam Nyon sampled in August 2018 and March 2019 (n = 175). Regression lines are shown for each village, with the shaded areas representing 95% confidence intervals. Pearson correlation r = −0.21 for Na Savang (n = 123, p = 0.021) and r = −0.67 for Nam Nyon (n = 52, p < 0.001). Figure S4: Distribution of δ 13C and δ 15N values (‰) of rice samples (n = 5). Sampling villages are indicated by the labeled boxes adjacent to each data point. Figure S5: Distribution of δ 13C and δ 15N values (‰) of terrestrial animal samples (n = 20). Table S3: Comparison of food consumption frequency (days of intake in the past week) between males from Na Savang and Nam Nyon surveyed in March 2019 (dry season). Table S4: Linear correlation between hair δ 13C and δ 15N values (‰) of males from Na Savang and Nam Nyon sampled in August 2018 (rainy season) and March 2019 (dry season). Table S5: Comparisons of household‐ and individual‐level variables among the subsistence clusters identified by the hierarchical cluster analysis in Nam Nyon (Figure S3). Table S6: Comparisons of isotopic, anthropometric, socioeconomic, and food consumption frequency data for males from Na Savang who participated in both August 2018 (rainy season) and March 2019 (dry season) surveys (n = 51). Table S7: Associations between hair δ 13C (‰) and socioeconomic variables in Na Savang, stratified by [file AJPA-190-e70326-s002.pdf]

Model		Variable	Nam Nyon	Na Savang	(Note)
**Models 1 & 2**		Age	√	√	Covariate
**Model 1: Livelihood**<br>Social, subsistence/economic		Ethnicity	√	√	Sociodemographic
	Subsistence/ Economic variable(s)	Subsistence cluster	√		Farmland; cash income
		Occupation		√	Subsistence activity
		Landholding		√	Stock; flow
		Rice self-sufficiency		√	Flow
		Roofing material		√	Stock (long-term)
		Possession index		√	Stock (short-term)
		Sampling season		√	
**Model 2: Diet**<br>Food consumption frequency		Wild terrestrial plant	√	√	
		Riverweed/algae	√	√	
		Cultivated C_3 plant	√	√	
		Local terrestrial animal	√	√	
		Local aquatic animal	√	√	
		Alcohol	√	√	
		Oil/fat	√	√	Binary
		Other commercial food	√	√	

Figure S1. Independent variables in the multilinear regression models for each village. The "subsistence cluster" refers to household categories obtained from a hierarchical cluster analysis based on farmland area and cash income (Figure S2). The "stock" variables represent long-term assets, whereas the "flow" variables indicate short-term income sources.

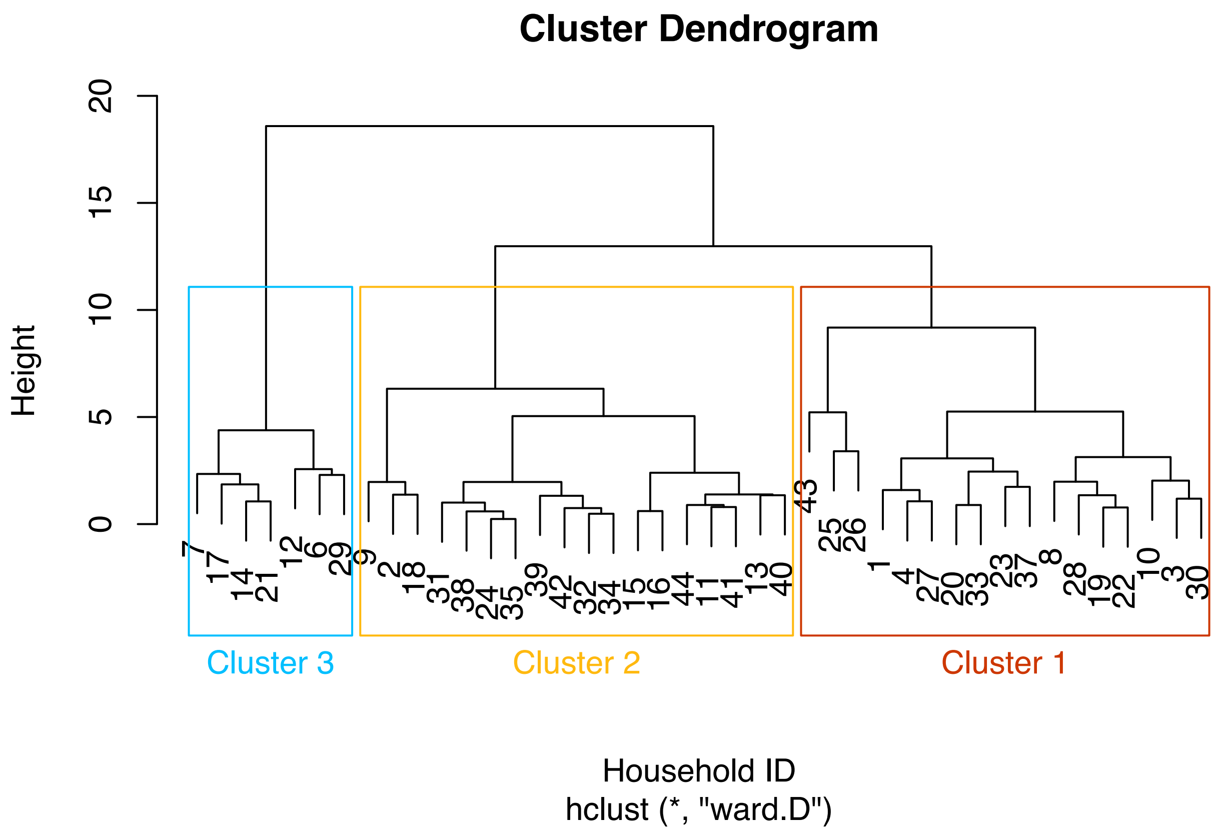

Figure S2. Clustering of 42 households in Nam Nyon by subsistence portfolios (Ward, 1963), using farmland area and cash income as clustering variables (see Table S5).

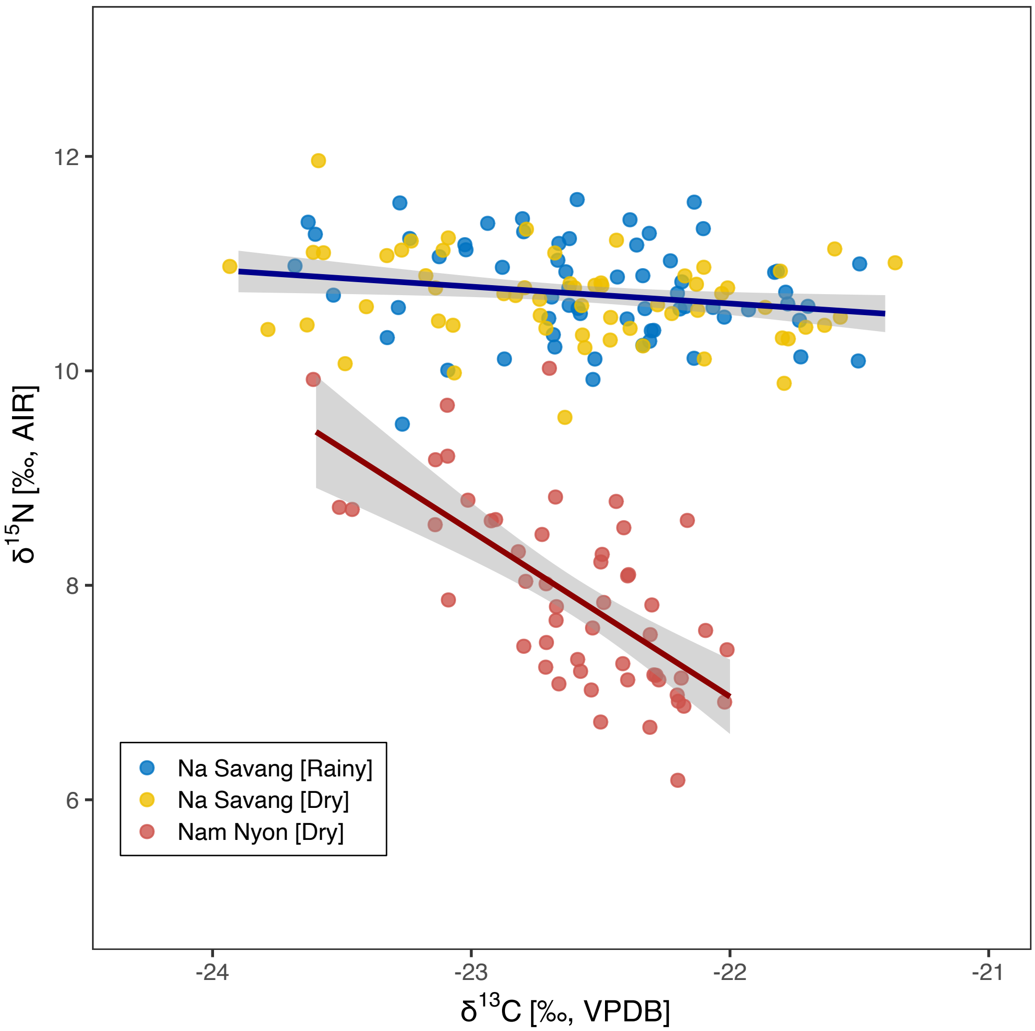

Figure S3. Linear correlation between hair δ^{13}C and δ^{15}N values (‰) of male individuals from Na Savang and Nam Nyon sampled in August 2018 and March 2019 (n = 175). Regression lines are shown for each village, with the shaded areas representing 95% confidence intervals. Pearson correlation $r = -0.21$ for Na Savang (n = 123, p = 0.021) and $r = -0.67$ for Nam Nyon (n = 52, $p < 0.001$).

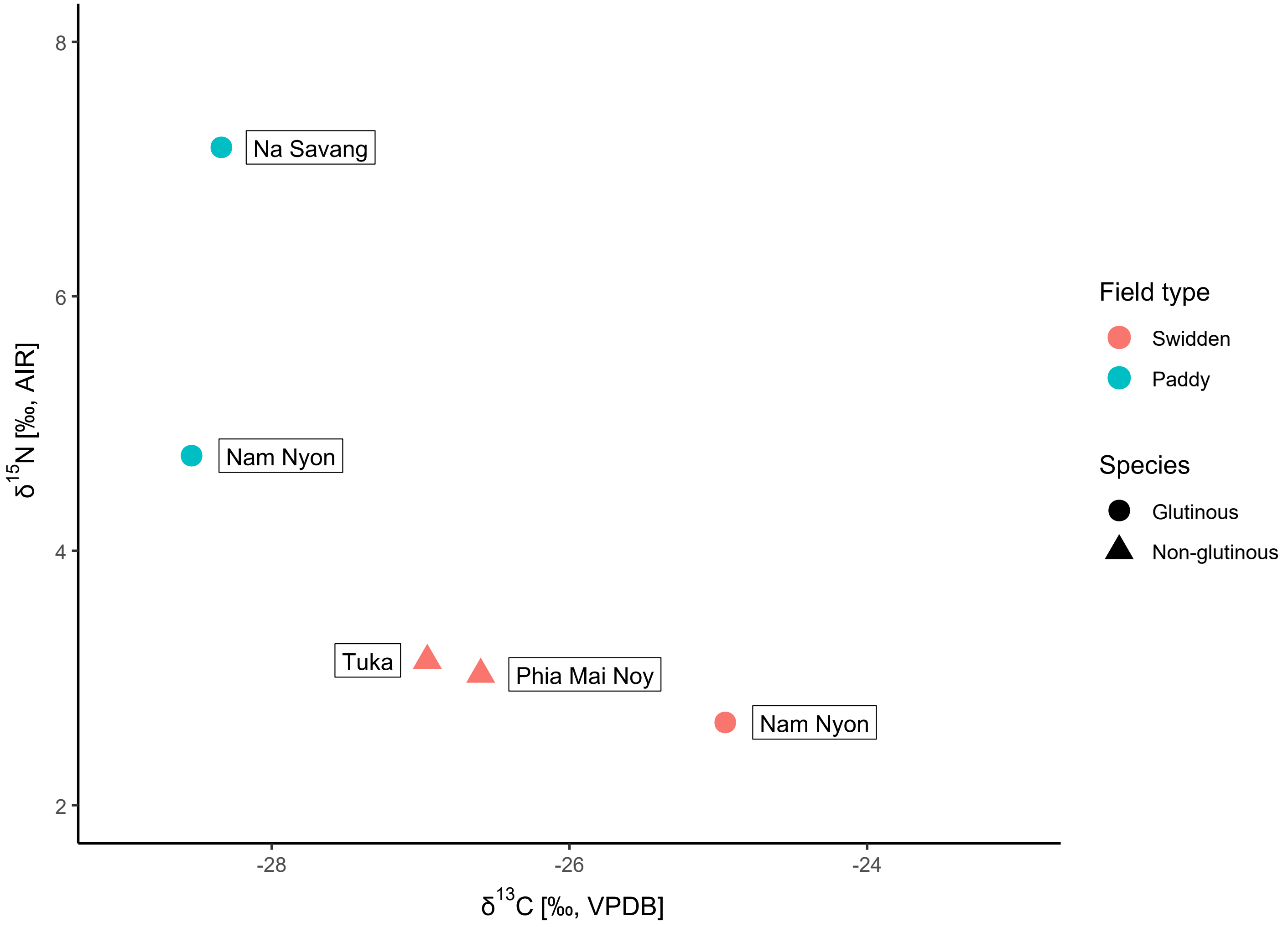

Figure S4. Distribution of $\delta^{13}C$ and $\delta^{15}N$ values (‰) of rice samples (n = 5). Sampling villages are indicated by the labeled boxes adjacent to each data point.

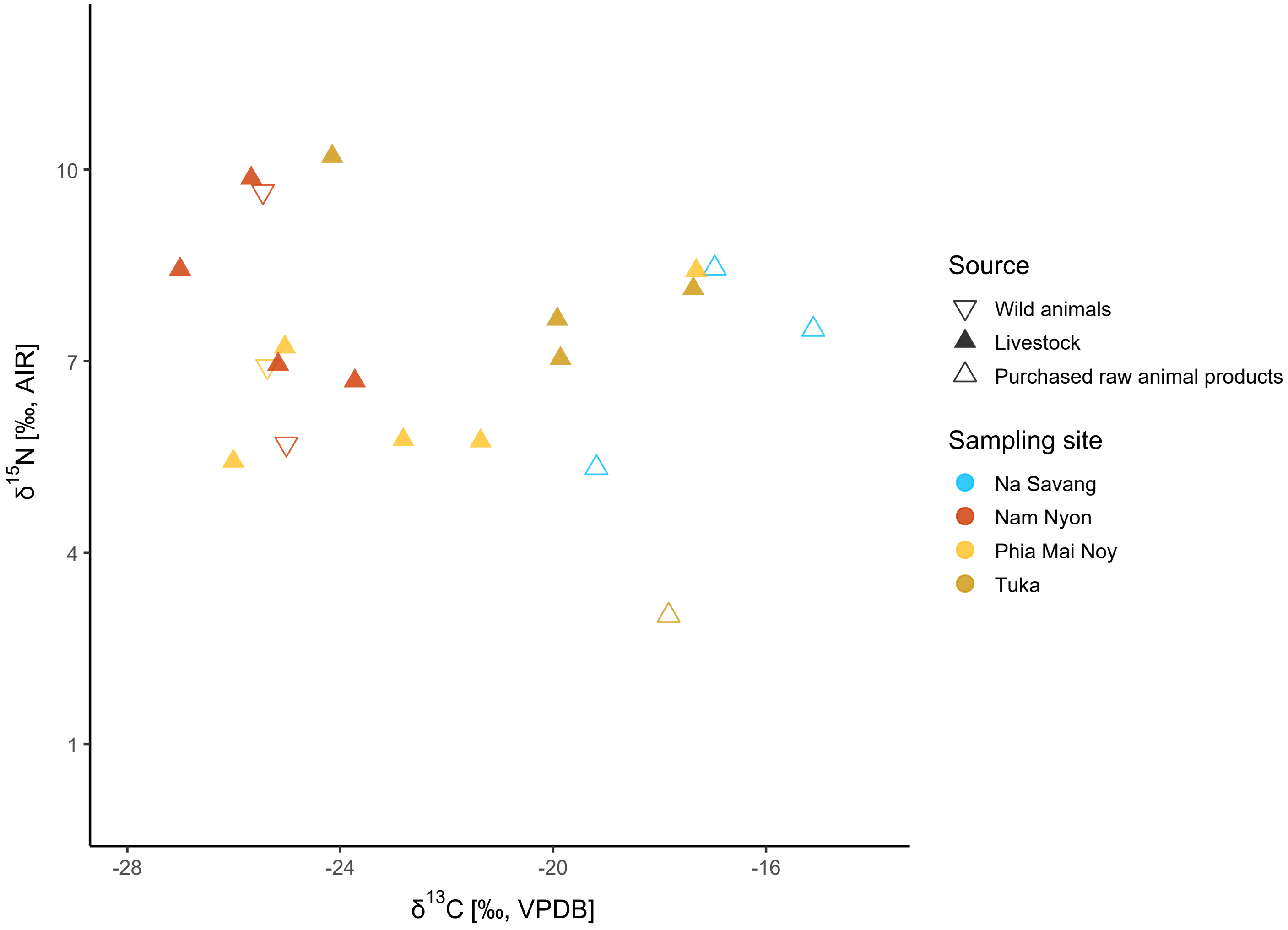

Figure S5. Distribution of δ^{13}C and δ^{15}N values (‰) of terrestrial animal samples (*n* = 20).

Table S3. Comparison of food consumption frequency (days of intake in the past week) between males from Na Savang and Nam Nyon surveyed in March 2019 (dry season).

	**Na Savang [Dry]**	**Nam Nyon [Dry]**	***p*-value**
N	59	52	
Wild terrestrial plants (max)	2 (1, 3)	3 (2, 4)	< 0.001
Bitter bamboo shoot	1 (0, 2)	2 (1, 3)	< 0.001
Other wild vegetables	2 (1, 3)	3 (2, 3)	0.001
Wild fruits	0 (0, 0)	0 (0, 1)	0.013
Riverweed/algae	3 (2, 4)	2 (2, 3)	0.13
Cultivated C_3 plants (max)	3 (2, 4)	5 (4, 7)	< 0.001
Tuber crops	0 (0, 2)	0 (0, 1)	0.6
Cultivated leafy vegetables	2 (0, 4)	4 (3, 7)	< 0.001
Cultivated fruits	1 (0, 2)	0 (0, 1)	< 0.001
Herbs	0 (0, 0)	0 (0, 0)	0.9
Rice	7 (7, 7)	7 (7, 7)	
Corn	0 (0, 1)	0 (0, 0)	< 0.001
Spices	7 (4, 7)	7 (4, 7)	0.2
Local terrestrial animals (max)	3 (2, 4)	2 (2, 3)	0.002
Meats	2 (1, 2)	0 (0, 1)	< 0.001
Poultry/eggs	2 (1, 3)	2 (1, 2)	0.5
Bushmeats	0 (0, 2)	1 (0, 2)	0.7
Insects/larvae	0 (0, 0)	0 (0, 0)	0.029
Animal organs	1 (0, 2)	0 (0, 1)	0.024
Animal blood	1 (0, 2)	1 (0, 2)	0.5
Fermented animal products	2 (1, 3)	1 (0, 2)	0.003
Local aquatic animals (max)	3 (2, 4)	3 (2, 3)	0.7
Fish	3 (1, 3)	2 (2, 3)	0.6
Fish organs	1 (0, 2)	1 (0, 1)	0.096
Other aquatic animals	2 (0, 2)	2 (2, 3)	0.008
Alcohol	1 (0, 2)	1 (0, 2)	> 0.9
Oil/fat	5 (2, 5)	4 (2, 7)	0.4
Other commercial foods (max)	3 (2, 3)	2 (2, 2)	0.007
Coffee/tea	0 (0, 2)	0 (0, 0)	< 0.001
Rice noodles	2 (1, 3)	0 (0, 1)	< 0.001
Processed meats	0 (0, 0)	0 (0, 0)	0.4
Canned fish	0 (0, 1)	0 (0, 1)	0.9
Sweets/snacks	1 (0, 2)	2 (1, 2)	0.2

The data are shown as median (interquartile range) and were compared using the Wilcoxon rank-sum test. The items without “(max)” indicate directly surveyed food items, whereas those labeled “(max)” represent aggregated categories based on isotopic characteristics, with values corresponding to the maximum within each category.

Table S4. Linear correlation between hair $\delta^{13}C$ and $\delta^{15}N$ values (‰) of males from Na Savang and Nam Nyon sampled in August 2018 (rainy season) and March 2019 (dry season).

	**Pearson's *r***	**(95% CI)**	***p*-value**
Na Savang (all)	−0.208	(−0.372, −0.032)	0.021
Na Savang [Rainy]	−0.189	(−0.415, 0.060)	0.135
Na Savang [Dry]	−0.252	(−0.478, 0.004)	0.054
Nam Nyon [Dry]	−0.666	(−0.795, −0.481)	< 0.001

CI = confidence interval.

Table S5. Comparisons of household- and individual-level variables among the subsistence clusters identified by the hierarchical cluster analysis in Nam Nyon (Figure S3).

	Cluster 1	Cluster 2	Cluster 3	Overall p	Grp diff.
Household subsistence variables					
N	17	18	7		
Land area (ha) [a]					
Swidden [b]	2 (2, 3)	1 (1, 2)	0.5 (0, 1)	< 0.001	*†‡
Paddy field	0.3 (0, 0.6)	0 (0, 0.3)	1.5 (1, 1.5)	< 0.001	†‡
Cash income (10,000 LAK) [c]					
Crop [d]	1,032 ± 513	401 ± 262	1,341 ± 430	< 0.001	*‡
NTFP [a]	157 (85, 240)	78 (35, 120)	45 (35, 125)	0.033	
Livestock [a]	30 (0, 50)	0 (0, 10)	110 (90, 230)	0.008	†‡
Individual-level variables					
N	24	20	6		
Hair δ^{13}C (‰) [a]	−22.5 (−22.7, −22.3)	−22.5 (−22.7, −22.2)	−23.1 (−23.5, −22.9)	0.003	†‡
Hair δ^{15}N (‰) [a]	7.8 (7.4, 8.4)	7.2 (7.0, 8.0)	8.8 (8.7, 9.7)	< 0.001	†‡
Age (years) [a]	31 (26, 42)	28 (25, 41)	40 (38, 44)	0.2	
Ethnicity [e]				0.018	*†
Kongsat	6 (25)	12 (60)	5 (83)		
Phunyot	17 (71)	8 (40)	1 (17)		
Other	1 (4.2)	0 (0)	0 (0)		
Rice self-sufficiency [e]				0.2	
Self-sufficient	15 (63)	17 (85)	4 (67)		
Surplus	9 (38)	3 (15)	2 (33)		
Food consumption frequency [a]					
Wild terrestrial plant	3 (2, 3)	4 (3, 4)	3 (3, 3)	0.2	
Riverweed/algae	2 (2, 4)	2 (1, 2)	3 (2, 3)	0.050	
Cultivated C_3 plant	6 (4, 7)	4 (4, 7)	4 (3, 4)	0.2	
Local terrestrial animal	2 (2, 3)	2 (2, 3)	3 (2, 3)	0.5	
Local aquatic animal	3 (2, 4)	2 (2, 3)	3 (2, 3)	0.093	
Alcohol	1 (0, 2)	1 (0, 2)	2 (1, 2)	0.5	
Oil/fat	7 (3, 7)	3 (2, 7)	7 (2, 7)	0.3	
Other commercial food	2 (2, 2)	2 (1, 3)	2 (2, 3)	0.7	

Grp diff. = group differences: significant differences between Clusters 1 and 2 (*), 1 and 3 (†), or 2 and 3 (‡) given by *post hoc* tests at $p < 0.05$.
[a] Median (interquartile range); Kruskal–Wallis test, with Dunn's *post hoc* test (Benjamini–Hochberg correction) when overall $p < 0.05$.
[b] The area of swiddens cultivated by households at the time of the field survey by Kibe et al. (2022) (i.e., in the rainy season of 2019), not the total area owned by households. Swiddens are farmed under a shifting cultivation system in which cultivated land is rotated annually.
[c] 10,000 LAK was equivalent to ~1.15 USD in 2019 and is worth ~0.46 USD as of September 2025.
[d] Mean ± standard deviation; one-way ANOVA, with Tukey's HSD test when overall $p < 0.05$.
[e] n (%); Fisher's exact test, with pairwise comparisons (Benjamini–Hochberg correction) when overall $p < 0.05$.

Table S6. Comparisons of isotopic, anthropometric, socioeconomic, and food consumption frequency data for males from Na Savang who participated in both August 2018 (rainy season) and March 2019 (dry season) surveys (n = 51).

	Na Savang [Rainy]	Na Savang [Dry]	p-value
Hair $\delta^{13}C$ (‰) [a]	−22.6 ± 0.6	−22.6 ± 0.6	0.2
Hair $\delta^{15}N$ (‰) [a]	10.7 ± 0.5	10.7 ± 0.4	0.2
Age (years) [b]	44 (36, 49)	44 (35, 52)	0.003
Household socioeconomic variables			
Landholding (ha) [b,c]	1.9 (1.0, 2.0)	1.8 (1.0, 2.8)	0.8
Occupation [d]			0.2
Only farming	33 (65)	39 (76)	
Only non-farming	1 (2)	0 (0)	
Both	17 (33)	12 (24)	
Concrete roof [e]			0.6
No	27 (53)	25 (49)	
Yes	24 (47)	26 (51)	
Possession index [b]	5 (4, 5)	5 (4, 5)	0.023
Food consumption frequency [b,f]			
Wild terrestrial plants (max)	2 (2, 3)	2 (1, 3)	0.4
Bitter bamboo shoot	0 (0, 0)	0 (0, 1)	0.042
Other wild vegetables	2 (1, 3)	2 (0, 3)	0.8
Wild fruits	1 (0, 2)	0 (0, 0)	< 0.001
Riverweed/algae	0 (0, 0)	3 (2, 5)	< 0.001
Cultivated C_3 plants (max)	3 (1, 5)	3 (2, 4)	0.8
Tuber crops	0 (0, 0)	0 (0, 1)	0.1
Cultivated leafy vegetables [c]	3 (0, 5)	3 (0, 4)	0.5
Cultivated fruits	1 (0, 3)	1 (0, 2)	0.7
Herbs	0 (0, 0)	0 (0, 0)	0.6
Rice	7 (7, 7)	7 (7, 7)	0.4
Corn	0 (0, 1)	0 (0, 1)	0.6
Spices	7 (5, 7)	7 (4, 7)	0.2
Local terrestrial animals (max)	3 (2, 4)	3 (2, 4)	0.6
Meats	2 (1, 3)	2 (1, 2)	0.062
Poultry/eggs	2 (1, 2)	2 (1, 2)	0.6
Bushmeats	0 (0, 1)	0 (0, 2)	0.032
Insects/larvae	0 (0, 0)	0 (0, 0)	1
Animal organs	0 (0, 1)	1 (0, 2)	0.2
Animal blood	1 (0, 2)	1 (0, 2)	0.5
Fermented animal products	1 (0, 2)	2 (1, 3)	0.1
Local aquatic animals (max)	3 (2, 5)	3 (2, 4)	0.7
Fish	3 (2, 4)	2 (1, 4)	0.5
Fish organs	0 (0, 1)	1 (0, 2)	0.012
Other aquatic animals	1 (0, 2)	2 (0, 2)	0.8
Alcohol	1 (0, 1)	1 (0, 2)	0.089

Oil/fat [c]	7 (4, 7)	5 (2, 7)	0.006
Other commercial foods (max)	2 (1, 3)	3 (2, 3)	0.8
Coffee/tea	0 (0, 1)	0 (0, 2)	0.6
Rice noodles	1 (1, 2)	2 (1, 3)	0.4
Processed meats	0 (0, 0)	0 (0, 0)	0.8
Canned fish	1 (0, 2)	0 (0, 1)	0.038
Sweets/snacks [c]	2 (0, 3)	1 (0, 2)	0.048

[a] Mean ± standard deviation; paired *t*-test.
[b] Median (interquartile range); Wilcoxon signed-rank test.
[c] 1 missing value in *Na Savang [Rainy]*.
[d] *n* (%); McNemar–Bowker test.
[e] *n* (%); McNemar's chi-squared test.
[f] The items without "(max)" indicate directly surveyed food items, whereas those labeled "(max)" represent aggregated categories based on isotopic characteristics, with values corresponding to the maximum within each category.

Table S7. Associations between hair $\delta^{13}C$ (‰) and socioeconomic variables in Na Savang, stratified by sampling season: Results of multiple linear regression analyses, adjusted for age.

		**Na Savang [Rainy]** [a]		**Na Savang [Dry]** [b]	
		b (95% CI)	*p*	*b* (95% CI)	*p*
Ethnicity	Yang (ref.)				
	Other	0.12 (−0.22, 0.46)	0.48	0.46 (0.04, 0.89)	0.03
Landholding (ha)		0.06 (−0.03, 0.15)	0.18	0.11 (−0.01, 0.23)	0.07
Occupation	Only farming (ref.)				
	Only non-farming	0.23 (−0.48, 0.94)	0.51	—	
	Both	−0.11 (−0.38, 0.16)	0.41	−0.22 (−0.61, 0.17)	0.27
Rice self-sufficiency	Self-sufficient (ref.)				
	Surplus	0.22 (−0.04, 0.48)	0.09	0.31 (0.01, 0.61)	0.04
Concrete roof	No (ref.)				
	Yes	0.20 (−0.05, 0.44)	0.12	0.28 (−0.03, 0.59)	0.08
Possession index		0.12 (−0.02, 0.26)	0.09	0.09 (−0.09, 0.28)	0.31
**Adjusted R^2**			0.22		0.24

b = unstandardized regression coefficient; CI = confidence interval; ref. = reference.
[a] $N = 62$ (2 deleted due to missingness)
[b] $N = 58$ (1 deleted due to missingness)
